# Double overexpression of DREB and PIF transcription factors improves drought stress tolerance and cell elongation in transgenic plants

**DOI:** 10.1111/pbi.12644

**Published:** 2016-11-14

**Authors:** Madoka Kudo, Satoshi Kidokoro, Takuya Yoshida, Junya Mizoi, Daisuke Todaka, Alisdair R. Fernie, Kazuo Shinozaki, Kazuko Yamaguchi‐Shinozaki

**Affiliations:** ^1^ Graduate School of Agricultural and Life SciencesUniversity of TokyoTokyoJapan; ^2^ Max‐Planck‐Institut für Molekulare PflanzenphysiologieGolmGermany; ^3^ RIKEN Center for Sustainable Resource ScienceTsurumi‐kuYokohamaJapan

**Keywords:** Dehydration‐Responsive Element‐Binding protein 1A (DREB1A), Rice Phytochrome‐Interacting factor‐Like 1 (OsPIL1), Drought stress tolerance, Cell elongation, Flowering, Arabidopsis

## Abstract

Although a variety of transgenic plants that are tolerant to drought stress have been generated, many of these plants show growth retardation. To improve drought tolerance and plant growth, we applied a gene‐stacking approach using two transcription factor genes: *DEHYDRATION‐RESPONSIVE ELEMENT‐BINDING 1A* (*DREB1A*) and rice *PHYTOCHROME‐INTERACTING FACTOR‐LIKE 1* (*OsPIL1*). The overexpression of *DREB1A* has been reported to improve drought stress tolerance in various crops, although it also causes a severe dwarf phenotype. *OsPIL1* is a rice homologue of Arabidopsis *PHYTOCHROME‐INTERACTING FACTOR 4* (*PIF4*)*,* and it enhances cell elongation by activating cell wall‐related gene expression. We found that the OsPIL1 protein was more stable than PIF4 under light conditions in Arabidopsis protoplasts. Transactivation analyses revealed that DREB1A and OsPIL1 did not negatively affect each other's transcriptional activities. The transgenic plants overexpressing both *OsPIL1* and *DREB1A* showed the improved drought stress tolerance similar to that of *DREB1A* overexpressors. Furthermore, double overexpressors showed the enhanced hypocotyl elongation and floral induction compared with the *DREB1A* overexpressors. Metabolome analyses indicated that compatible solutes, such as sugars and amino acids, accumulated in the double overexpressors, which was similar to the observations of the *DREB1A* overexpressors. Transcriptome analyses showed an increased expression of abiotic stress‐inducible DREB1A downstream genes and cell elongation‐related OsPIL1 downstream genes in the double overexpressors, which suggests that these two transcription factors function independently in the transgenic plants despite the trade‐offs required to balance plant growth and stress tolerance. Our study provides a basis for plant genetic engineering designed to overcome growth retardation in drought‐tolerant transgenic plants.

## Introduction

Drought is one of the most serious environmental stresses that affect global agriculture. In recent years, prolonged droughts have caused severe damage to crops in many of the most agriculturally productive areas of the world. At present, approximately 800 million people are undernourished (FAO, IFAD and WFP, 2015). In addition, the world population is expected to experience dramatic grow; thus, the world food crisis appears to be worsening. Therefore, to ensure a stable supply of foods and biomass materials, it is imperative to improve drought stress tolerance in crops.

Plant growth is repressed in response to environmental stresses, indicating that there are trade‐offs between growth and stress tolerance (Claeys and Inze, 2013). Although a variety of transgenic plants that are tolerant to drought stress have been generated, many of these plants show growth retardation (Yamaguchi‐Shinozaki and Shinozaki, 2006). To reduce the negative effects on plant growth, stress‐inducible promoters have been used to drive the expression of transgenes in transgenic plants (Bhatnagar‐Mathur *et al*., 2007; Kasuga *et al*., 1999; Pino *et al*., 2007; Suo *et al*., 2012). However, growth retardation appears to be unavoidable under the long‐term drought stress conditions because of the prolonged overexpression of transgenes, even when using stress‐inducible promoters. Additionally, it is necessary to select the optimal stress‐inducible promoters for each plant species. For example, an Arabidopsis *RESPONSIVE TO DEHYDRATION 29A* (*RD29A*) promoter is useful for overexpression during abiotic stress conditions in both Arabidopsis and tobacco, whereas it functions in the roots, but not the leaves, in rice (Ito *et al*., 2006; Kasuga, 2004). Therefore, additional approaches are required to improve the growth of stress‐tolerant plants.

Pyramiding (gene‐stacking) breeding is essential for biotechnology applications (Halpin, 2005). For example, plants that produce several *Bacillus thuringiensis* (Bt) proteins have been shown to improve insect resistance (Carriere *et al*., 2015). ‘Golden rice’ plants similarly contain three carotenoid biosynthesis genes for accumulating pro‐vitamin A (Ye *et al*., 2000). The co‐expression of 9‐cis‐epoxycarotenoid dioxygenase (NCED) and D‐arabinono‐1,4‐lactone oxidase (ALO) increases abscisic acid (ABA) and ascorbic acid levels and improves tolerance to drought and chilling *in planta* (Bao *et al*., 2016). Many genetically modified (GM) crops cultivated worldwide have been generated by gene stacking (Halpin, 2005). However, few studies have focused on engineering plants to improve both drought stress tolerance and growth using gene‐stacking approaches.

DREB1A is an APETALA2/ethylene‐responsive element‐binding factor (AP2/ERF)‐type transcription factor that specifically binds dehydration‐responsive elements (DREs) and up‐regulates stress‐inducible target gene expression. The overexpression of *DREB1A* improves the tolerance to drought, salt and freezing stress by enhancing late embryogenesis‐abundant (LEA) protein levels and the compatible solute contents in Arabidopsis (Kasuga *et al*., 1999; Maruyama *et al*., 2004, 2009). Overexpression of the Arabidopsis *DREB1A* gene was reported to enhance abiotic stress tolerance in many crops, such as rice, soybean, peanut and wheat (Bhatnagar‐Mathur *et al*., 2014; Ito *et al*., 2006; Pellegrineschi *et al*., 2004; Suo *et al*., 2012). Moreover, the mechanism of improved drought stress tolerance was revealed by transcriptome and metabolome analyses as presented above. Therefore, *DREB1A* appears to be one of the most agriculturally useful genes for improving abiotic stress tolerance in crops. However, *DREB1A* overexpression causes dwarfism and late flowering in plants, and additional investigations are required before it can be used in efficient agricultural applications.

Arabidopsis phytochrome‐interacting factor (PIF) family proteins, which are basic helix‐loop‐helix (bHLH)‐type transcription factors, were initially isolated through their interaction with phytochrome (Ni *et al*., 1999). PIFs function to promote seedling skotomorphogenesis, shade avoidance and floral induction and regulate the expression of many downstream genes (Leivar and Quail, 2011). In rice, six PIF‐like genes were identified by *in silico* analysis (Nakamura *et al*., 2007). Of these, OsPIL1 was reported to enhance cell elongation through the activation of cell wall synthesis‐related genes in rice (Todaka *et al*., 2012). Additionally, *OsPIL1* expression levels are repressed under drought and low‐temperature conditions; thus, OsPIL1 appears to act as a key regulator of plant growth in response to abiotic stress.

In this study, to overcome the trade‐offs between growth and stress tolerance, we generated transgenic plants overexpressing both *OsPIL1* and *DREB1A* and characterized these double overexpressors by phenotypic analyses, metabolome analyses and genomewide transcriptome analyses. We propose that *OsPIL1* partially enhances plant growth and accelerates flowering time, even in the double overexpressors, without negative effects on DREB1A‐mediated drought stress tolerance.

## Results

### Stability of the rice OsPIL1 protein and transactivation activity of OsPIL1 with DREB1A in Arabidopsis and rice protoplasts

Arabidopsis PIF family proteins have been reported to enhance cell elongation, and their protein levels are regulated by light stimulation (Leivar and Quail, 2011). Light triggers the interaction of PIF family proteins with phytochrome B (PhyB), which results in the degradation of the PIF proteins by the 26S proteasome. However, we previously reported that one of the four amino acid residues important for PhyB binding in Arabidopsis PIF proteins is not conserved in the rice PIF, including protein OsPIL1, which does not interact with OsPhyB in yeast two‐hybrid and bimolecular fluorescence complementation (BiFC) assays (Todaka *et al*., 2012). Thus, we analysed the OsPIL1 protein stability in Arabidopsis mesophyll protoplasts under light conditions. sGFP‐fused OsPIL1 and PIF4 proteins were transiently expressed in Arabidopsis protoplasts, and the levels of these proteins were then analysed by immunoblotting (Figure S1). In addition, 3 × Flag‐tagged sGFP was co‐expressed in protoplasts as an internal control. To determine whether the OsPIL1 protein is degraded under light conditions, we incubated the transfected protoplasts in the dark and then transferred the protoplasts to light conditions in the absence or presence of the 26S proteasome inhibitor MG132 (Figure S1a). In the absence of MG132, the PIF4 protein was degraded in a light‐ and time‐dependent manner as previously reported (Nozue *et al*., 2007), whereas the OsPIL1 protein was more stable under the same conditions (Figure S1b). In addition, PIF4 and OsPIL1 were not degraded in the presence of MG132 (Figure S1b). These results demonstrate that OsPIL1 is more stable than PIF4 and show that OsPIL1 does not appear to be degraded by the 26S proteasome in response to light irradiation. Taken together, these results suggest that OsPIL1 may enhance plant growth more effectively than PIF4 in Arabidopsis under light conditions.

**Figure 1:**
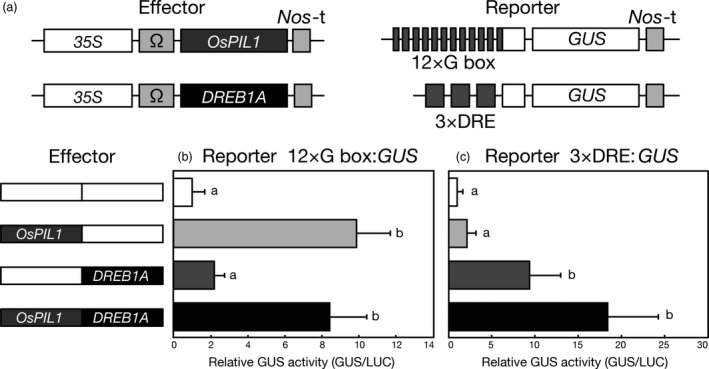
Effect of co‐expressing both OsPIL1 and DREB1A on each transactivation activity in Arabidopsis protoplasts. (a) Schematic diagram of the effector and reporter constructs used in the transactivation analysis of the Arabidopsis protoplasts. The effector construct contains a *CaMV 35S* promoter and a TMV Ω sequence fused to the coding sequence of *OsPIL1* or *DREB1A*. *Nos*‐t indicates the polyadenylation signal of the gene for nopaline synthetase. The reporter construct 12 × G box:*GUS* contains 12 tandem 28‐bp G box‐containing fragments. The reporter construct 3 × DRE:*GUS* contains three tandem 71‐bp DRE‐containing fragments. (b, c) Transactivation effects of the co‐expression of OsPIL1 and DREB1A. The reporter 12 × G box:*GUS* (b) or 3 × DRE:*GUS* (c) and the effectors were co‐transfected into Arabidopsis protoplasts. To normalize for transfection efficiency, an *emerald luciferase* (*ELUC*) reporter gene driven by the *CaMV 35S* promoter was co‐transfected as a control in each experiment. Bars show the standard deviation (SD) of more than four replicates. The letters indicate significant differences among the assays (*P* < 0.05 according to Games–Howell's multiple range test).

OsPIL1 and DREB1A have been reported to recognize the *cis*‐acting promoter elements G box (CACGTG) and DRE (A/GCCGAC), respectively (Liu *et al*., 1998; Todaka *et al*., 2012). To examine whether these transcription factors affect each other's transactivation activity, we performed transactivation assays using Arabidopsis and rice mesophyll protoplasts (Figures 1 and S2). *35S*Ω:*OsPIL1* and *35S*Ω:*DREB1A* were used as effector constructs, and G box:*GUS* or DRE:*GUS* was used as a reporter construct (Liu *et al*., 1998; Todaka *et al*., 2012). The expression of *OsPIL1* with the G box:*GUS* reporter construct showed high transactivation activity (Figures 1b and S2b). Similarly, when both *OsPIL1* and *DREB1A* were co‐expressed, high transactivation activity was observed. When DRE:*GUS* was used as the reporter, the transactivation activity was enhanced by the co‐expression of both OsPIL1 and DREB1A in a manner similar to the expression of DREB1A (Figures 1c and S2c). These results indicate that OsPIL1 and DREB1A act independently and do not interfere with each other's transactivation activities in Arabidopsis and rice protoplasts.

### Enhanced cell elongation and improved drought stress tolerance in transgenic plants overexpressing both *OsPIL1* and *DREB1A* (double overexpressors)

**Figure 2:**
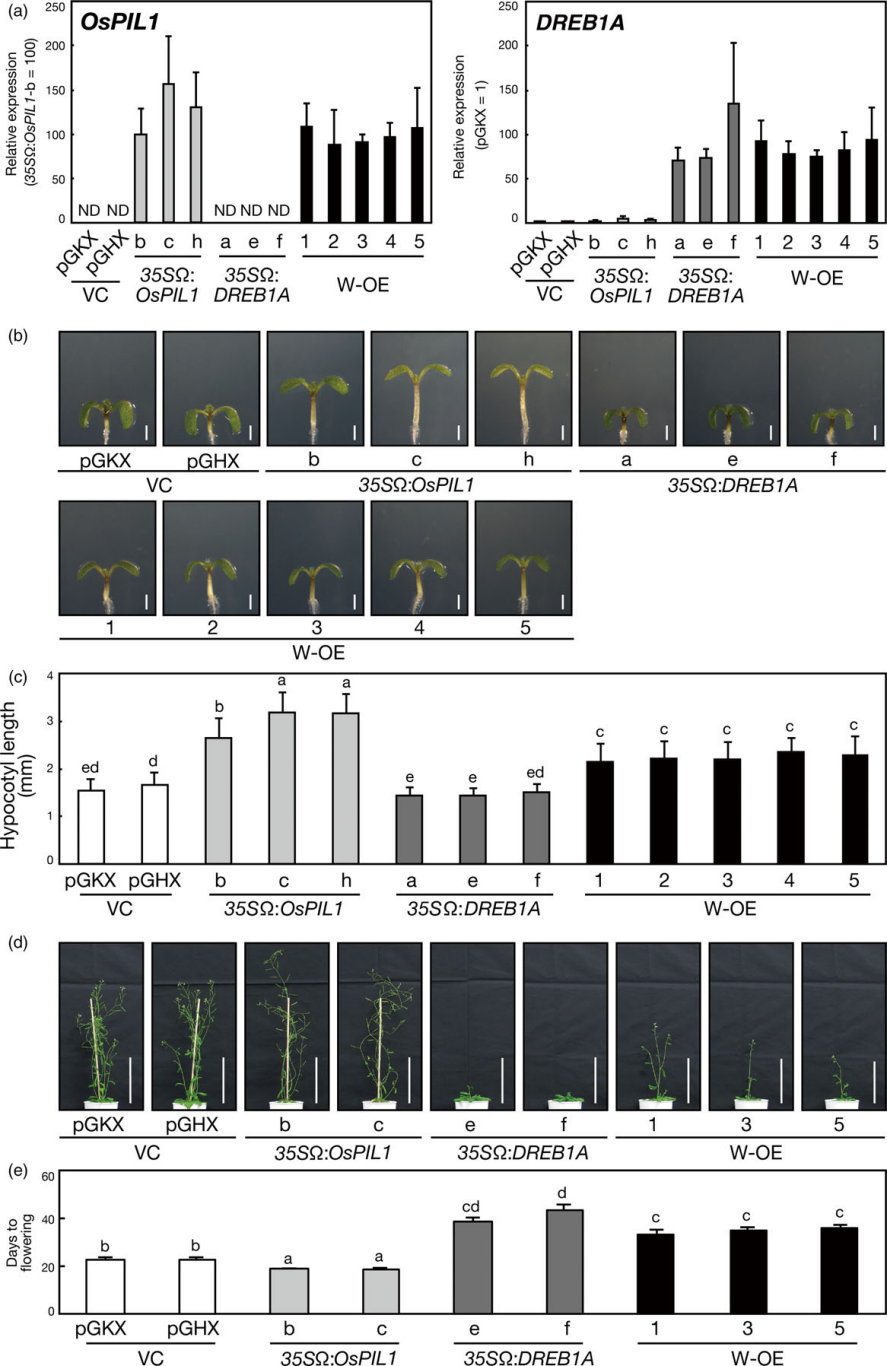
Generation of *OsPIL1* and *DREB1A* double‐overexpressing Arabidopsis plants. (a) Expression levels of the transgenes in the single‐ or double‐overexpressing plants were analysed by quantitative RT‐PCR. The plants were grown on agar medium for 2 weeks. The error bars indicate the SD of more than four samples. ND represents not detected and VC and W‐OE represent vector control and *OsPIL1 DREB1A* double overexpressor, respectively. (b) Morphology of the transgenic Arabidopsis seedlings. The plants were grown on agar medium for 7 days. Bars = 1 mm. (c) Hypocotyl length calculated from the seedlings grown as in (b). The error bars show the SD of more than 50 seedlings. The letters indicate significant differences among the seedlings (*P* < 0.01 according to Games–Howell's multiple range test). (d, e) Flowering time of the single‐ or double‐overexpressing Arabidopsis plants. (d) Growth of 6‐week‐old transgenic plants. The plants were grown on agar medium for 2 weeks and then in soil pots. Bars = 10 cm. (e) Days to flowering of the plants grown as in (d). The error bars show the SD of more than four plants. The letters indicate significant differences among the plants (*P* < 0.05 according to Games–Howell's multiple range test).

Next, we generated transgenic Arabidopsis plants overexpressing both *OsPIL1* and *DREB1A* (double overexpressors) by crossing *DREB1A* overexpressors with *OsPIL1* overexpressors (Figure 2a, Table S1). *OsPIL1* or *DREB1A* was overexpressed under the control of the *CaMV 35S* promoter and the TMV Ω sequence in transgenic plants. To explore whether the double overexpressors have phenotypes related to the overexpression of each *OsPIL1* and *DREB1A*, we observed the morphology of these transgenic plants. Overexpression of rice *OsPIL1* was reported to enhance hypocotyl elongation in Arabidopsis (Nakamura *et al*., 2007; Todaka *et al*., 2012). Thus, we measured the hypocotyl length of 7‐day‐old double overexpressors compared with that of *35S*Ω:*OsPIL1* and *35S*Ω:*DREB1A* plants (Figure 2b, c). Significant differences were not observed in hypocotyl length between the two vector control plants, pGKX and pGHX (Figure 2c). The hypocotyl length of the double overexpressors was intermediate between the *35S*Ω:*OsPIL1* and the vector control plants. The hypocotyl length of the *35S*Ω:*DREB1A* plants was equivalent to that of the vector control plants. We also observed the lengths of the hypocotyl cells in these transgenic plants and found that hypocotyl length correlated with cell length (Figure S3). In addition, we found that the internode and shoot lengths of 8‐week‐old double overexpressors were longer than those of the *35S*Ω:*DREB1A* plants (Figures S4 and S5). These results indicate that overexpressing *OsPIL1* enhances cell elongation of the hypocotyl and stem, even in the double overexpressors, and show that overexpressing *DREB1A* has a negative effect on OsPIL1‐mediated cell elongation. Furthermore, *DREB1A* overexpression has been reported to improve drought stress tolerance while causing a severe dwarf phenotype (Liu *et al*., 1998). Under normal growth conditions, the double overexpressors also showed small rosette leaves and short primary roots, which is similar to the *35S*Ω:*DREB1A* plants (Figure S6a, b, c, d). Next, we weighed the total dry biomass of the transgenic Arabidopsis plants. The double overexpressors had a dry weight similar to the *35S*Ω:*DREB1A* plants (Figure S6e). In addition, we evaluated the seed yield of the plants. Significant differences were not observed in average silique number between the vector control, *35S*Ω:*DREB1A* and double overexpressor plants, but this number was decreased in the *35S*Ω:*OsPIL1* plants compared with the other plants (Figure S6f). The average seed number per silique was similar in all the transgenic plants (Figure S6g). Therefore, overexpression of *OsPIL1* decreased the seed yield of the transgenic plants. This may be due to the early flowering and senescence phenotypes of the *35S*Ω:*OsPIL1* plants. Overexpression of Arabidopsis *PIF4* is also known to induce early senescence in transgenic plants (Sakuraba *et al*., 2014). However, overexpression of *OsPIL1* did not affect the seed yield in the double overexpressors.

In addition to growth retardation*,* the overexpression of *DREB1A* causes late‐flowering phenotypes in transgenic Arabidopsis plants (Seo *et al*., 2009), whereas the overexpression of *PIF4*, an Arabidopsis homologue of *OsPIL1*, causes an extremely early flowering phenotype by elevating *FLOWERING LOCUS T* (*FT*) expression levels (Kumar *et al*., 2012). Therefore, we analysed the flowering time of the transgenic plants (Figure 2d, e). Compared with the vector control plants, the *35S*Ω:*OsPIL1* plants showed early flowering and the *35S*Ω:*DREB1A* plants showed late flowering. The flowering time of the double overexpressors was earlier than that of the *35S*Ω:*DREB1A* plants, but later than that of the vector control and *35S*Ω:*OsPIL1* plants. These results suggest that OsPIL1 and DREB1A antagonistically regulate flowering time in the double overexpressors. Thus, *OsPIL1* is expected to be useful for weakening the late‐flowering phenotypes caused by *DREB1A* overexpression. Taken together, the double overexpressors present phenotypes caused by the overexpression of each *OsPIL1* and *DREB1A*.

**Figure 3:**
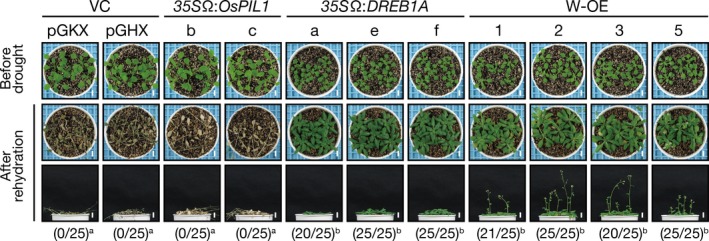
Drought stress tolerance of the single or double overexpressors. Number codes show the number of surviving plants out of the total number. A total of 25 plants were used in the survival test. Bars = 1 cm. The letters indicate significant differences among the plants (*P* < 0.05 according to Tukey's multiple range test of the ratio).

We then evaluated the drought stress tolerance of the double overexpressors by withholding water for 2 weeks. No improvement in drought stress tolerance was observed in the *35S*Ω:*OsPIL1* plants, whereas the double overexpressors showed the improved drought stress tolerance similar to that of the *35S*Ω:*DREB1A* plants (Figure 3), indicating that overexpressing *OsPIL1* has no negative effects on the DREB1A‐mediated improvement of drought stress tolerance in the double overexpressors.

### OsPIL1 and DREB1A affect sugar and amino acid metabolism in the double overexpressors

**Figure 4:**
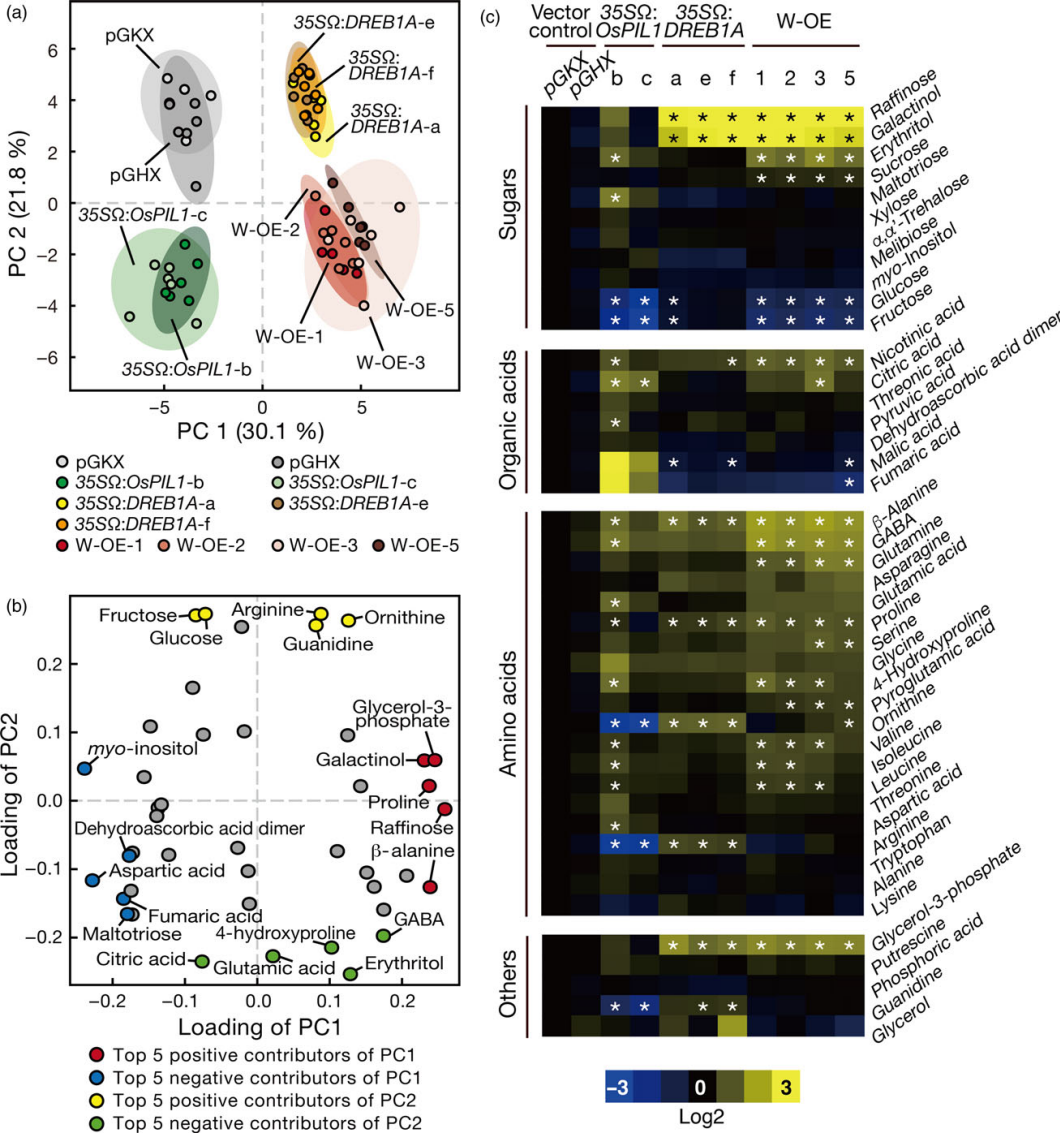
Metabolite profiles of the single‐ or double‐overexpressing plants. (a, b) Principal components analysis (PCA) of metabolite profile in the transgenic plants. Score plot (a) and loading plot (b) of the metabolite profiles in the transgenic plants. The plants were grown on agar medium for 2 weeks and harvested at Zeitgeber time (ZT) = 0. GABA, 4‐aminobutyric acid. (c) Heat maps of the metabolite profile in the transgenic plants. The relative metabolite amounts were normalized with vector control plants (pGKX) and transformed to log2. Yellow and blue colours show increased and decreased levels, respectively. Asterisks indicate significant differences between the vector control plants (both pGKX and pGHX) (*P* < 0.05 according to Tukey's multiple range test, n = 6).

To reveal the metabolite profiles of the double overexpressors, we performed gas chromatography–time‐of‐flight mass spectrometry (GC‐TOF‐MS) analyses of 2‐week‐old (vegetative stage) transgenic plants and identified 43 metabolites, including sugars, amino acids and organic acids (Figures 4, S7a). We extracted the soluble metabolites from whole plants harvested at the subjective dawn (Zeitgeber time [ZT] = 0) and the subjective sundown (ZT = 16). Because the metabolite profiles of the double overexpressors harvested at these two sampling points were nearly equivalent, the differences in the metabolite profiles among the tested transgenic plants were independent of their daily changes (Figure S7b, c). According to principal components analysis (PCA), the contribution ratios of the first (PC1) and second principal components (PC2) were 30.1% and 21.8%, respectively (Figure 4a). The *35S*Ω:*DREB1A* and *35S*Ω:*OsPIL1* plants were separated from the vector control plants by PC1 and PC2. Interestingly, the metabolite profiles of the double overexpressors were clearly different from those of each single overexpressor. The score plot of the double overexpressors was described by the metabolites that contributed to the positive side of the PC1 axis and the negative side of the PC2 axis (Figure 4a). We then compared the loadings of PC1 and PC2 to clarify the metabolites that contributed to the separation of the PCA score (Figure 4b). The top five metabolites contributing to the positive side of PC1 were raffinose, glycerol‐3‐phosphate, β‐alanine, proline and galactinol, and those contributing to the negative side were *myo*‐inositol, aspartic acid, fumaric acid, maltotriose and dehydroascorbic acid dimer. However, the top five positive contributors to PC2 were glucose, arginine, fructose, ornithine and guanidine, and the top five negative contributors were erythritol, citric acid, glutamic acid, 4‐hydroxyproline and 4‐aminobutyric acid (GABA).

In the *35S*Ω:*DREB1A* plants, the amounts of raffinose, galactinol, β‐alanine, proline, ornithine, arginine and glycerol‐3‐phosphate were significantly increased relative to the vector control plants (Figure 4c). Galactinol and raffinose have been reported to act as osmoprotectants in drought stress tolerance in plants (Taji *et al*., 2002). Proline and β‐alanine are precursors of quaternary ammonium compounds (QAC), which also act as osmoprotectants (Hanson *et al*., 1994). Following the overexpression of *OsPIL1*, the amount of citric acid was increased, whereas the amounts of glucose, fructose, ornithine, arginine and guanidine were decreased relative to the vector control plants (Figure 4c). The metabolism of ornithine and arginine appeared to be oppositely affected by the overexpression of *DREB1A* and *OsPIL1*, and the levels of these metabolites in the double overexpressors were the average of those in the single overexpressors. The amounts of GABA and glutamine were slightly increased in the *35S*Ω:*DREB1A* plants and clearly increased in the double overexpressors (Figure 4c). These results suggest that both OsPIL1 and DREB1A independently regulate sugar and amino acid metabolism in the double overexpressors.

### Downstream genes of both OsPIL1 and DREB1A were activated in the double overexpressors

**Figure 5:**
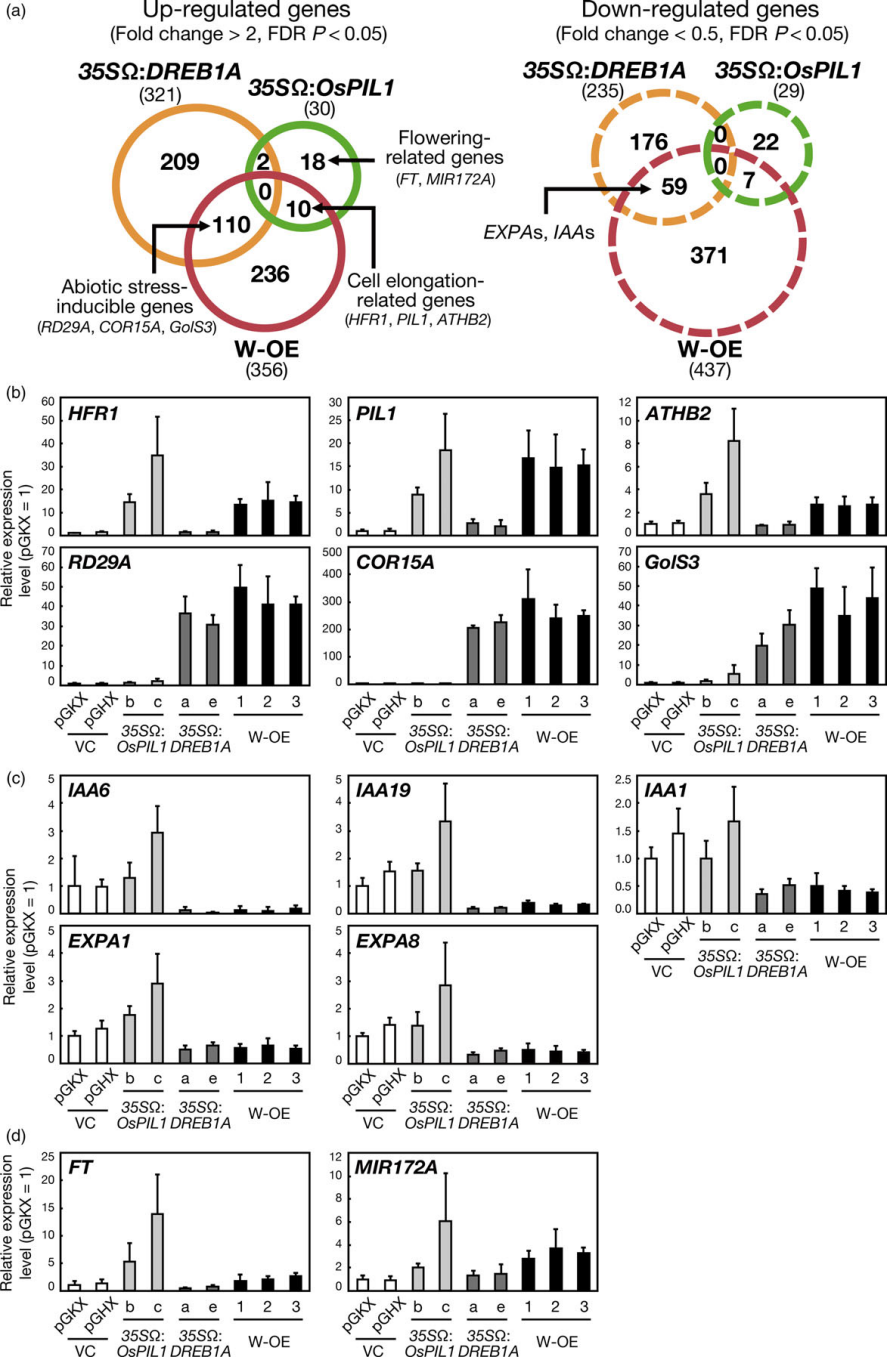
Comparative analyses of the transcriptomes of single‐ or double‐overexpressing plants. (a) Venn diagrams comparing up‐regulated and down‐regulated genes among the *35S*Ω:*DREB1A* plants (orange circles), the *35S*Ω:*OsPIL1* plants (green circles) and the double overexpressors (red circles). The plants were grown on agar medium for 2 weeks and harvested at ZT = 0. Up‐regulated and down‐regulated genes are in solid circles and dashed circles, respectively. The total numbers of up‐regulated and down‐regulated genes are shown in parentheses. (b, c, d) Expression levels of up‐regulated genes (b), down‐regulated genes (c) and flowering regulator genes (d) in the transgenic Arabidopsis plants were analysed by quantitative RT‐PCR. The plants were grown on agar medium for 2 weeks and harvested at ZT = 0. The error bars indicate the SD of more than four samples.

To reveal the transcript profiles, we performed microarray experiments using 2‐week‐old *35S*Ω:*OsPIL1* plants and double overexpressors harvested at ZT = 0. The overexpression of *OsPIL1* alone and *OsPIL1* and *DREB1A* in combination up‐regulated the expression of 30 and 356 genes, respectively (fold change > 2, false discovery rate [FDR] *P* < 0.05) (Figure 5a, left). We compared these up‐regulated genes with those in the *35S*Ω:*DREB1A* plants (Kidokoro *et al*., 2015) and found 110 common genes between the *35S*Ω:*DREB1A* plants and the double overexpressors, and these genes included many abiotic stress‐inducible genes (Table S2). Ten genes overlapped between the *35S*Ω:*OsPIL1* plants and the double overexpressors, including many cell elongation‐related genes (Table S3). No genes overlapped among all three transgenic Arabidopsis plants. The number of down‐regulated genes in the *35S*Ω:*DREB1A* plants, *35S*Ω:*OsPIL1* plants and double overexpressors was 235, 29 and 437, respectively (fold change < 0.5, FDR *P* < 0.05) (Figure 5a, right). Fifty‐nine genes were common between the *35S*Ω:*DREB1A* plants and the double overexpressors, including cell wall‐related genes (Table S4). Seven genes overlapped between the *35S*Ω:*OsPIL1* plants and the double overexpressors, although the functions of these genes were not relevant (Table S5). We observed many up‐regulated or down‐regulated genes in the double overexpressors, but not in the *35S*Ω:*OsPIL1* or *35S*Ω:*DREB1A* plants alone. However, the majority of these genes were either up‐regulated or down‐regulated at low levels. The fold change values for approximately 70% of the up‐regulated or down‐regulated genes were less than threefold or more than 0.4‐fold (Tables S6 and S7).

Next, we analysed the expression patterns of up‐regulated and down‐regulated genes in each transgenic Arabidopsis plant using the microarray database at Genevestigator (https://genevestigator.com/gv/). The downstream genes in the double overexpressors were responsive to ABA, cold, drought, osmotic and salt stress, which is similar to the effects in the *35S*Ω:*DREB1A* plants (Figure S8). Certain up‐regulated genes in the *35S*Ω:*OsPIL1* plants were responsive to ABA, indole‐3‐acetic acid (IAA) and far‐red (FR) light (Figure S8). To obtain additional information on the molecular functions of the downstream genes, we categorized these genes according to overrepresentation analyses using PageMan software (http://mapman.mpimp-golm.mpg.de/). Compared with the categorization of the complete Arabidopsis genome, the up‐regulated genes in the *35S*Ω:*DREB1A* plants showed higher ratios of the categories secondary metabolism (12.7%), stress (11.7%), hormone metabolism (6.4%) and sugar metabolism (4.5%) (Figure S9). The ratios of the genes in the categories RNA (27.1%), hormone metabolism (12.9%) and signalling (8.6%) were increased in the *35S*Ω:*OsPIL1* plants (Figure S9). The ratios of the genes in the categories hormone metabolism (13.6%) and cell wall (5.0%) were higher among the down‐regulated genes in the *35S*Ω:*DREB1A* plants (Figure S10). Genes in the categories secondary metabolism (11.5%), sugar metabolism (6.4%) and cell wall (11.5%) were overrepresented among the down‐regulated genes in the *35S*Ω:*OsPIL1* plants (Figure S10). The ratios of each category of up‐regulated and down‐regulated genes in the double overexpressors were similar to the average of the values in the *35S*Ω:*OsPIL1* and *35S*Ω:*DREB1A* plants (Figures S9 and S10). Furthermore, because raffinose and galactinol were accumulated in the double overexpressors, sugar metabolism in the transgenic plants was analysed using the MapMan database (http://mapman.gabipd.org/). In the sugar metabolism pathway, genes for galactinol synthases and raffinose synthase were up‐regulated in *35S*Ω:*DREB1A* and the double overexpressors (Figure S11). However, a gene for sucrose invertase was down‐regulated in *35S*Ω:*OsPIL1* (Figure S11).

We confirmed the expression levels of the downstream genes in the transgenic plants by using quantitative RT‐PCR (Figure 5b, c, d). *LONG HYPOCOTYL IN FAR‐RED* (*HFR1*), *PHYTOCHROME‐INTERACTING FACTOR 3‐LIKE 1* (*PIL1*) and *ARABIDOPSIS THALIANA HOMEOBOX PROTEIN 2* (*ATHB2*) are cell elongation‐related target genes of PIF4 in Arabidopsis (Hornitschek *et al*., 2012; Kunihiro *et al*., 2011). The expression levels of these three genes increased in the *35S*Ω:*OsPIL1* plants and the double overexpressors (Figure 5b). *RD29A*,* COLD‐REGULATED 15A* (*COR15A*) and *GALACTINOL SYNTHASE 3* (*GolS3*) are major abiotic stress‐inducible targets of DREB1A (Maruyama *et al*., 2009), and the expression of these genes was elevated in the *35S*Ω:*DREB1A* plants and the double overexpressors (Figure 5b). These up‐regulated genes may be important for cell elongation and drought stress tolerance in the double overexpressors. However, *INDOLE‐3‐ACETIC ACID‐INDUCIBLE* (*IAA*) genes and *EXPANSIN A* (*EXPA*) genes were down‐regulated in the *35S*Ω:*DREB1A* plants and the doble overexpressors (Figure 5c). *IAA* genes are auxin inducible and act as repressors in auxin signalling (Reed, 2001). *EXPA*s are known to enhance cell wall enlargement by cell wall loosening (Cosgrove, 2000). In addition, we analysed the expression levels of flowering‐related genes, such as *FT* and *MICRORNA172A* (*MIR172A*), in the transgenic plants (Figure 5d). *FT* encodes florigen, whose overexpression causes early flowering (Turck *et al*., 2008). Overexpression of *MIR172A* also causes early flowering by down‐regulating AP2‐like floral repressors (Aukerman and Sakai, 2003). The expression levels of *FT* and *MIR172A* were elevated in the *35S*Ω:*OsPIL1* plants (Figure 5d). However, the expression levels of *FT* in the *35S*Ω:*DREB1A* plants were approximately half of those in the vector control plants. In the double overexpressors, the expression levels of *FT* and *MIR172A* were higher than those in the control plants, which suggests that OsPIL1 enhances floral induction by regulating *FT* and *MIR172A* expression in the transgenic plants.

Furthermore, we analysed the expression levels of other PIF4 downstream genes, namely *XYLOGLUCAN ENDOTRANSGLYCOSYLASE 7* (*XTR7*), *PACLOBUTRAZOL RESISTANCE1* (*PRE1*) and *IAA29* (Hornitschek *et al*., 2012; de Lucas *et al*., 2008; Nomoto *et al*., 2012; Oh *et al*., 2012); however, significant differences were not observed in the *35S*Ω:*OsPIL1* plants and double overexpressors compared with the vector control plants (Figure S12a). These results suggest that certain OsPIL1 target genes differ from those of PIF4. We then analysed the expression levels of *SMALL AUXIN‐UPREGULATED RNA* (*SAUR*) genes (Figure S12b). SAURs positively regulate cell elongation to promote hypocotyl growth, and PIF4 has been shown to up‐regulate the expression of *SAUR19‐24* at high temperatures (Franklin *et al*., 2011; Ren and Gray, 2015). The expression levels of *SAUR19, SAUR22* and *SAUR23* were elevated in the *35S*Ω:*OsPIL1* plants and the double overexpressors (Figure S12b), indicating that OsPIL1 also enhances cell elongation through the up‐regulation of *SAUR* genes, which is similar to the action of PIF4 in the transgenic Arabidopsis plants. These results suggest that OsPIL1 acts as a positive regulator of auxin signalling.

**Figure 6:**
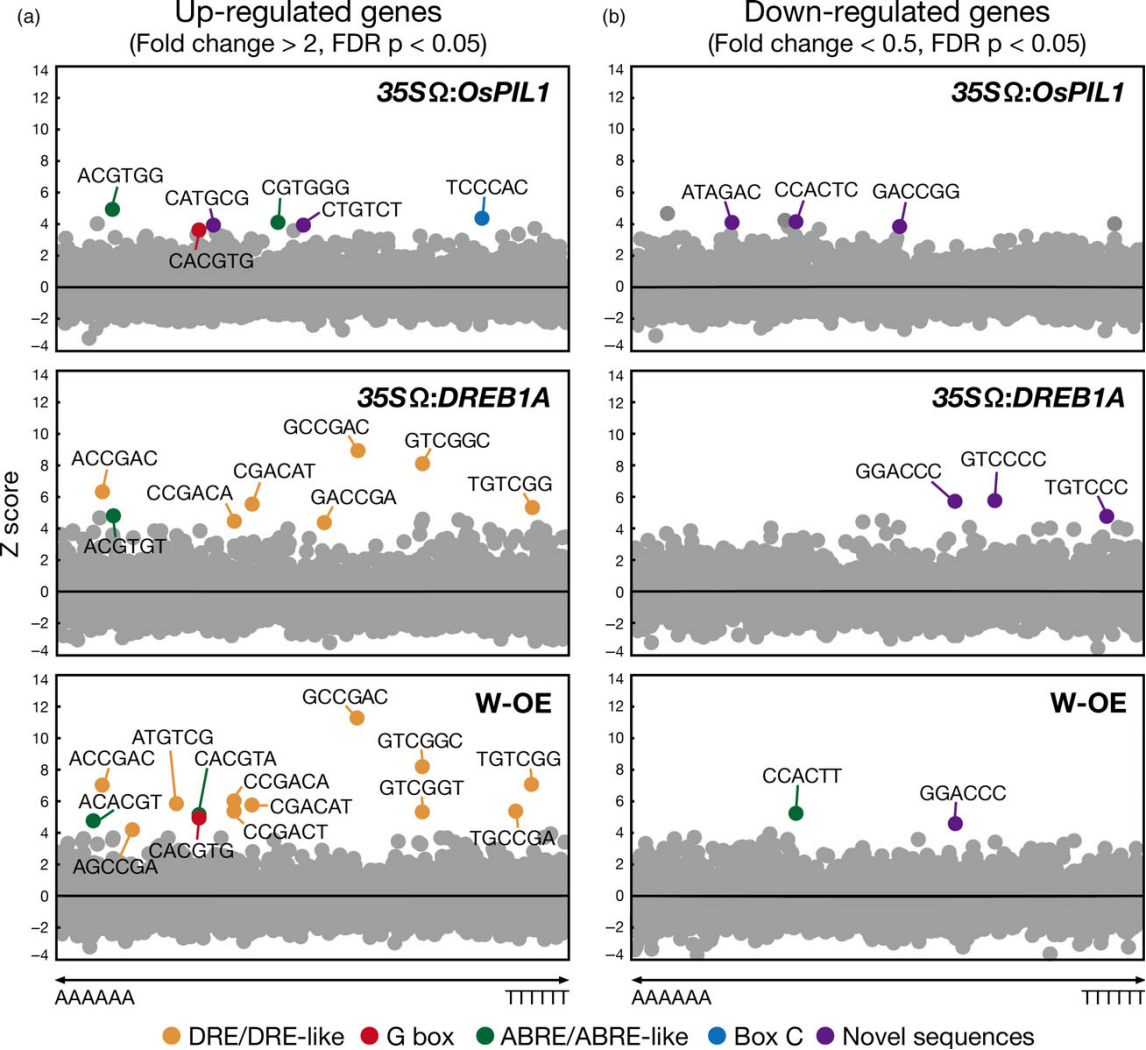
Overrepresentation analyses of hexamer sequences in the promoters of up‐regulated and down‐regulated genes in the single or double overexpressors. Scatter plots showing Z scores (*y*‐axes) for the observed frequencies of all hexamer sequences (*x*‐axes) in the 1‐kb promoters of up‐regulated (a) and down‐regulated genes (b) in each transgenic plant. DRE/DRE‐like (orange), G box (red), ABRE/ABRE‐like (green), Box C (blue) and novel sequences (purple) are shown.

To characterize the promoters of the up‐regulated and down‐regulated genes in the double overexpressors, we assessed the enrichment of all hexamer motifs (from AAAAAA to TTTTTT, 4^6^ = 4096) in the promoters of the genes in each transgenic plant (Figure 6). We compared the frequency of each hexamer sequence in the promoters of the genes with their normalized frequencies in Arabidopsis promoters (Maruyama *et al*., 2012). In the *35S*Ω:*OsPIL1* plants, G box (CACGTG) and ABRE sequences (ACGTGG and CGTGGG) ranked among the top 10 up‐regulated genes (Figure 6a, top). For the *35S*Ω:*DREB1A* plants, we analysed microarray data from a previous study (Figure 6a, middle; Kidokoro *et al*., 2015). Consistent with previous work, the most overrepresented sequence in the *35S*Ω:*DREB1A* plants was DRE (Maruyama *et al*., 2012). In the double overexpressors, DRE sequences occupied the top 10, and G box or ABRE sequences were ranked within the top 15 among the up‐regulated genes (Figure 6a, bottom). The double overexpressor‐specific elements were not found in the promoter regions of the up‐regulated genes. These results indicate that OsPIL1 and DREB1A activate the expression of each downstream gene independently via each *cis*‐acting element in the double overexpressors. However, known *cis* elements were not identified among the enriched hexamer motifs in the promoters of the down‐regulated genes in the *35S*Ω:*OsPIL1* plants and the double overexpressors (Figure 6b). In the *35S*Ω:*DREB1A* plants, known hexamer motifs were also not observed, suggesting that *IAA* and *EXPA* gene expression is indirectly repressed by DREB1A.

## Discussion

Considerable effort has been devoted to producing GM plants with improved tolerance to abiotic stress, such as drought and high salinity, by using molecular breeding strategies. The overexpression of *DREB1A* induces many stress‐inducible target genes and enhances drought stress tolerance in various crops, although it also causes the growth retardation (Liu *et al*., 1998). To improve drought stress tolerance and plant growth, we generated transgenic plants overexpressing two transcription factors: DREB1A and OsPIL1. OsPIL1 is reported to be a homologue of Arabidopsis PIF4 and acts as a key regulator of internode elongation in rice by up‐regulating many cell elongation‐related genes, including cell wall‐related genes (Todaka *et al*., 2012). We observed that the overexpression of *OsPIL1* also up‐regulated several cell elongation‐related genes and increased hypocotyl and stem lengths in Arabidopsis plants (Figures 2b, c, 5b, S3, S4 and S5). The double overexpressors showed improved drought stress tolerance and enhanced hypocotyl elongation compared with the vector control plants (Figures 2b, c and 3), suggesting that plant genetic engineering using gene‐stacking approaches will be useful for generating transgenic plants that exhibit improved drought stress tolerance and enhanced growth.

The results of our transcriptome and metabolome analyses of the transgenic plants supported the idea that the two transcription factors function additively in the double overexpressors (Figures 4, 5 and 6). Regarding the improvement of drought stress tolerance, we observed an accumulation of compatible solutes, such as galactinol and raffinose, and the up‐regulation of the corresponding biosynthesis genes in the double overexpressors (Figures 4c and S11, Table S2). Because these data were also reported in the *DREB1A* overexpressors (Maruyama *et al*., 2009), the drought stress tolerance of the double overexpressors appears to be increased via the same mechanisms in the *DREB1A* overexpressors. *OsPIL1* overexpression increased the amount of citric acid and decreased the amount of glucose, fructose, ornithine, arginine and guanidine, and similar alterations were observed in the amounts of these metabolites in the double overexpressors (Figure 4c). Transcriptome analyses indicated that the expression levels of many downstream genes of OsPIL1 and DREB1A were also up‐regulated in the double overexpressors (Figures 5b and S12b). The major downstream genes of the two transcription factors (*RD29A*,* COR15A*,* GolS3* and *PIL1*) were up‐regulated by more than 20‐fold (Tables S2 and S3), while the expression levels of the up‐regulated and down‐regulated genes only in the double overexpressors were low (Tables S6 and S7). Our data suggest that cross‐regulation among the downstream genes may not be critical in the double overexpressors. In addition, we found that both the G box and DRE (which target the *cis*‐acting elements of OsPIL1 and DREB1A, respectively) were enriched in the promoters of the up‐regulated genes (Figure 6a). These results indicate that OsPIL1 and DREB1A act additively on the promoters of each target gene and suggest that these two transcription factors are unlikely to negatively affect each other in the transgenic plants. Thus, the co‐expression of OsPIL1 does not affect the DREB1A‐mediated improvement of drought stress tolerance in the double overexpressors, indicating that *OsPIL1* has the potential to improve the growth of transgenic plants tolerant to abiotic stress.

A late‐flowering phenotype was one of the negative effects of *DREB1A* overexpression in Arabidopsis (Figure 2d, e), and it has also been reported in other *DREB1*‐overexpressing plants, such as soybean and barley (Gilmour *et al*., 2004; Morran *et al*., 2011; Seo *et al*., 2009; Suo *et al*., 2012), indicating that *DREB1* overexpression results in a prolonged vegetative stage and delays the harvest time of crops. In the present study, we demonstrated that the delayed flowering time of *DREB1A*‐overexpressing plants was improved by *OsPIL1* co‐expression through the up‐regulation of the floral inducers *FT* and *MIR172A* (Figures 2d, e and 5d; Aukerman and Sakai, 2003; Turck *et al*., 2008). Thus, the appropriate expression of these genes is important for controlling flowering time in agricultural applications, and *OsPIL1* overexpression can be used to improve the delayed flowering times caused by transgenic *DREB1A* overexpression.

Arabidopsis PIF4 is known to promote shade avoidance syndrome (SAS), which consists of hypocotyl elongation, petiole elongation, stem elongation and hyponasty (Franklin *et al*., 2011; Hornitschek *et al*., 2012; Koini *et al*., 2009; Kumar *et al*., 2012), through the up‐regulation of auxin‐related genes (*IAA*s and *SAUR*s), shade‐inducible genes (*ATHB2*,* PIL1* and *HFR1*) and cell wall‐related genes (*XTR7*) (Casal, 2013). We showed that the overexpression of rice *OsPIL1* enhanced the hypocotyl and stem elongation in Arabidopsis (Figures 2b, c, S3 and S4). In the double overexpressors, *HFR1*,* PIL1*,* ATHB2* and *SAUR* mRNA levels were increased (Figures 5b and S12b). These results indicate that cell elongation in the *35S*Ω:*OsPIL1* plants is mainly caused by the elevated expression of SAS‐related genes similar to PIF4. Despite the similar expression levels of SAS‐related genes in the double overexpressors (Figure 5b), the hypocotyl length was shorter than that of the *35S*Ω:*OsPIL1* plants, suggesting that *DREB1A* overexpression negatively affects the hypocotyl cell elongation by regulating the expression of genes other than SAS‐related genes.

The function of PIF family proteins in plant metabolic pathways has not been reported. In this study, we observed higher levels of citric acid and lower levels of glucose, fructose, ornithine and arginine in the *35S*Ω:*OsPIL1* plants compared with the vector control plants (Figure 4c). Higher or lower levels of these primary metabolites have also been reported in the *pseudo response regulator* (*prr*) *9‐11 prr7‐10 prr5‐10* triple mutant (*d975*) of Arabidopsis (Fukushima *et al*., 2009). PRR9, 7 and 5 are known to function as transcriptional repressors in the circadian clock and repress the expression of *PIF* genes (Nakamichi *et al*., 2012). The *d975* mutant shows an accumulation of *PIF* transcripts and hypocotyl elongation (Nakamichi *et al*., 2009). Therefore, PIF family proteins may act to maintain primary plant metabolism under the regulation of circadian clock components. According to our transcriptome data, the gene expression level of *CELL WALL INVERTASE 5* (*CWINV5*), an enzyme that degrades sucrose into glucose and fructose, was decreased in the *35S*Ω:*OsPIL1* plants (Figure S11). This reduction in *CWINV5* expression partly accounted for the decrease in fructose and glucose. Our results suggest that PIF family proteins function in the regulation of primary metabolism.

Compared with the hypocotyl and stem, the rosette leaf size and dry weight in the double overexpressors were not increased and remained similar to those in the *35S*Ω:*DREB1A* plants (Figure S6a, b, e). The overexpression of *OsPIL1* has been shown to enhance the internode elongation, but not flag leaf length, in rice (Todaka *et al*., 2012). Therefore, the overexpression of *OsPIL1* might not affect the phenotypes of rosette leaves in the double overexpressors. We found that the *EXPA* gene expression levels decreased in the *35S*Ω:*DREB1A* plants and double overexpressors (Figure 5c). The negative regulation of *EXPA* genes may be a cause of the dwarfed rosette leaves in the *35S*Ω:*DREB1A* plants and double overexpressors.

We demonstrated that *OsPIL1* overexpression partially enhances the plant growth in transgenic plants that overexpress *OsPIL1* and *DREB1A*. The co‐overexpression of two transcription factors is considered an effective strategy for plant genetic engineering designed to improve drought stress tolerance and plant growth. In addition, gene‐stacking approaches appear to be able to tilt the balance in favour of growth in abiotic stress‐tolerant transgenic plants. We expect that the growth of stress‐tolerant plants will be further enhanced by the use of stress‐specific promoters that control transgene expression. Moreover, as the rosette leaf size in the double overexpressors was not increased, the utilization of genes encoding other growth regulators for leaf expansion may be an effective strategy for increasing the biomass and yield of stress‐tolerant plants. Our study provides a basis for overcoming growth retardation in stress‐tolerant plants through genetic engineering by gene stacking.

## Experimental procedures

### Plant materials and growth conditions

Arabidopsis (*Arabidopsis thaliana*) ecotype Columbia plants were grown on Murashige–Skoog (MS) agar medium at 22°C under a 16‐h/8‐h light/dark cycle at a photon flux density of 40 ± 10 μmol/m^2^/s as previously described (Yamaguchi‐Shinozaki and Shinozaki, 1994).

### Protein stability analysis in protoplasts

A protein stability analysis using the protoplasts derived from Arabidopsis mesophyll cells was performed according to Mizoi *et al*. (2013) with minor modifications. OsPIL1‐sGFP and PIF4‐sGFP were expressed under the control of the *CaMV 35S* promoter and TMV Ω sequence. Approximately 1.6 × 10^5^ protoplasts were transformed with 33.25 μg of OsPIL1‐sGFP or PIF4‐sGFP plasmid and 1.75 μg of 3 × FLAG‐sGFP. A 14‐h incubation at 22°C in the dark was followed by incubation in a solution containing 50 μm MG132 or 0.5% (v/v) dimethyl sulphoxide (DMSO, as a solvent control) under white‐light conditions (50 ± 5 μmol/m^2^/s). After 2 and 4 h, the protoplasts were precipitated by centrifugation and dissolved in 40 μL of sample buffer. The extracts were denatured at 95°C for 3 min and then analysed by SDS–PAGE and immunoblotting. The sGFP‐fused proteins were detected with a polyclonal antibody against GFP (Tanaka *et al*., 2012). Horseradish peroxidase‐conjugated anti‐rabbit IgG antibodies (Pierce, Rockford, IL), ECL Plus (GE Healthcare, Chalfont, UK) and an ImageQuant LAS 4000 system (GE Healthcare) were used to visualize the signals.

### Transient reporter assays with Arabidopsis and rice protoplasts

Transient expression assays using the protoplasts derived from Arabidopsis and rice mesophyll cells were performed as previously described (Kidokoro *et al*., 2009; Matsukura *et al*., 2010; Mizoi *et al*., 2013). The effector plasmids were constructed as described in Figures 1 and S2. Plasmids containing 12 tandem repeats of a 28‐bp G box‐containing fragment from the rice *1‐AMINOCYCLOPROPANE‐1‐CARBOXYLATE OXIDASE1* (*OsACO1*) promoter (G box:*GUS*) or three tandem repeats of a 71‐bp DRE‐containing fragment from the *RD29A* promoter (DRE:*GUS*) were used as reporters (Liu *et al*., 1998; Todaka *et al*., 2012). An *emerald luciferase* (*ELUC*) reporter gene driven by the *CaMV 35S* promoter (*35S*Ω:*ELUC*) and a *luciferase* (*LUC*) reporter gene driven by the *maize ubiquitin* promoter (*Ubi*:*LUC*) were used as internal controls in the Arabidopsis and rice protoplasts, respectively (Matsukura *et al*., 2010; Mizoi *et al*., 2013).

### Construction of plasmids and generation of *OsPIL1* and *DREB1A* double‐overexpressing plants

We used pGKX and pGHX vectors as binary vectors (Fujita *et al*., 2012; Qin *et al*., 2008). The pGKX and pGHX vectors include genes that confer antibiotic resistance to kanamycin and hygromycin, respectively. To generate *OsPIL1* or *DREB1A* single overexpressors, the coding sequences were inserted into the backbone binary vectors to construct pGKX‐*35S*Ω:*OsPIL1* and pGHX‐*35S*Ω:*DREB1A* plasmids. Arabidopsis plants were transformed using the floral dip method (Clough and Bent, 1998). *OsPIL1* and *DREB1A* double‐overexpressing Arabidopsis plants were generated by crossing *35S*Ω:*DREB1A* plants with *35S*Ω:*OsPIL1* plants (Table S1).

### Drought stress tolerance test

A dehydration treatment was performed as previously described (Liu *et al*., 1998) with minor modifications. The plants were grown on agar medium for 2 weeks and then in pots with soil for a week. Watering was withheld from 3‐week‐old plants for 2 weeks, and the survival rates were determined after 4 days of recovery following rehydration.

### Metabolome analysis

Metabolites were extracted from the whole plants grown on agar medium for 2 weeks. Extraction, derivatization and GC‐TOF‐MS analyses were performed as previously described (Lisec *et al*., 2006). The data were processed using TagFinder (Luedemann *et al*., 2008) and Xcalibur software (Thermo Scientific, Waltham, MA), and the relative amounts were normalized using ribitol as an internal standard and the fresh weight of the transgenic plants.

### Expression analysis

Total RNA was isolated from 2‐week‐old plants using RNAiso plus (TaKaRa Bio, Otsu, Japan) according to the manufacturer's instructions. cDNA synthesis and quantitative RT‐PCR were performed as previously described (Sato *et al*., 2014). The relative expression levels were calculated using the delta Ct method, and the obtained values were normalized to the *18S rRNA*. The primer sequences are shown in Table S8.

### Microarray experiment and data processing

Genome‐wide expression analyses using the Arabidopsis 3 Oligo Microarray were performed as previously described (Mizoi *et al*., 2013) with minor modifications. Normalization and statistical analyses of the data were performed using Subio Platform software (https://www.subio.jp/). The signal intensities were normalized by the Lowess method, and the significance of the expression changes was evaluated by one‐sample *t*‐tests. The p values were corrected by the Benjamini–Hochberg false discovery rate (FDR) method, and probes with an FDR of less than 0.05 were used for further analyses. All microarray data are available at Array Express under accession numbers E‐MTAB‐4073 and E‐MTAB‐4739.

## Supporting information


**Figure S1.** Stability of the OsPIL1 protein in Arabidopsis protoplasts under light conditions. (a) Schematic diagram of the treatments used in the assay. Transfected protoplast suspensions were treated with 0.5% dimethyl sulfoxide (DMSO) or 50 μM MG132 under white light (50 ± 5 μmol photons/m^2^/s). (b) Stability of the OsPIL1 and PIF4 proteins. OsPIL1 and PIF4 proteins were expressed as sGFP fusion proteins under the control of the *CaMV 35S* promoter and the TMV Ω sequence. To normalize for transfection efficiency, 3 × Flag‐tag fused‐sGFP driven by the *CaMV 35S* promoter was co‐transfected into the protoplasts as an internal control. The levels of the fusion proteins were analyzed by immunoblotting using an antibody against GFP.
**Figure S2.** Effect of co‐expressing both OsPIL1 and DREB1A on their individual transactivation activity in rice protoplasts. (a) Schematic diagram of the effector and reporter constructs used in the transactivation analysis of the rice protoplasts. The effector construct contains the *maize ubiquitin* promoter fused to the coding sequence of *OsPIL1* or *DREB1A*. (b, c) Transactivation effects of DREB1A and OsPIL1 co‐expression. The reporter 12 × G box:*GUS* (b) or 3 × DRE:*GUS* (c) and the effectors were co‐transfected into rice protoplasts. To normalize for transfection efficiency, the *luciferase* (*LUC*) reporter gene driven by the *maize ubiquitin* promoter was co‐transfected as a control in each experiment. Bars show the SD of 4 replicates. The letters indicate significant differences among the assays (*P* < 0.01 according to Games‐Howell's multiple range test).
**Figure S3.** Hypocotyl cell size of the single‐ or double‐overexpressing plants. (a) Cell surface in the hypocotyls of transgenic plants. The plants were grown on agar medium for 7 days. The cells were stained with toluidine blue. Bars = 50 μm. (b) Cell length calculated from the plants grown as in (a). The error bars show the SD of more than 50 cells in more than 4 seedlings (n > 200). The letters indicate significant differences among the seedlings (*P* < 0.01 according to Games‐Howell's multiple range test). VC and W‐OE represent vector control and *OsPIL1 DREB1A* double overexpressor, respectively.
**Figure S4.** Internode length of the single‐ or double‐overexpressing plants. (a) Stem morphology in 8‐week‐old transgenic plants. The plants were grown on agar medium for 2 weeks and then in soil pots for 6 weeks. Bars = 1 cm. (b) Internode length of the transgenic plants grown as in (a). The error bars show the SD of 10 internodes in 6 plants (n = 60). The letters indicate significant differences among the plants (*P* < 0.05 according to Games‐Howell's multiple range test).
**Figure S5.** Shoot length of the single‐ or double‐overexpressing plants. (a) Morphology of 8‐week‐old transgenic plants. The plants were grown on agar medium for 2 weeks and then in soil pots for 6 weeks. Bars = 10 cm. (b) Shoot length of the transgenic plants grown as in (a). The error bars show the SD of more than 4 plants. The letters indicate significant differences among the plants (*P* < 0.05 according to Games‐Howell's multiple range test).
**Figure S6.** Growth of the single‐ or double‐overexpressing plants. (a) Growth of 3‐week‐old transgenic Arabidopsis plants. The plants were grown on agar medium. Bars = 1 cm. (b) Average radius of the rosette calculated from the plants grown as in (a). The error bars show the SD of 14 plants. The letters indicate significant differences among the plants (*P* < 0.01 according to Tukey's multiple range test). (c) Growth of 12‐day‐old transgenic Arabidopsis plants on vertically oriented agar medium. Black bars show the root apex of the transgenic plants. White bar = 1 cm. (d) Average primary root length of the transgenic plants grown as shown in (c). The error bars show the SD of more than 4 plants. The letters indicate significant differences among the plants (*P* < 0.05 according to Tukey's multiple range test). (e) Average dry weight of 6‐week‐old transgenic Arabidopsis plants. The error bars show the SD of 6 plants. The letters indicate significant differences among the plants (*P* < 0.01 according to Games‐Howell's multiple range test). (f) Silique number in the transgenic Arabidopsis plants. The plants were grown on agar medium for 2 weeks and then in soil pots for more than 10 weeks. The error bars show the SD of more than 4 plants. The letters indicate significant differences among the plants (*P* < 0.05 according to Tukey's multiple range test). (g) Seed number per silique for the transgenic plants grown as shown in (f). The error bars show the SD of 5 siliques in more than 4 plants (n > 20).
**Figure S7.** Comparison of the metabolite profiles in the transgenic plants at different sampling time points. (a) Growth of 2‐week‐old transgenic Arabidopsis plants. The plants were grown on agar medium. Bars = 1 cm. Metabolite profiles of the transgenic plants harvested at the start of the day (ZT = 0) (b) and the end of the day (ZT = 16) (c). The relative amounts of the metabolites are displayed as heat maps. Yellow and blue colors show increased and decreased levels, respectively.
**Figure S8.** Hormone metabolism, abiotic stress, and light responses of the up‐regulated and down‐regulated genes in the single or double overexpressors. The expression ratios of the up‐regulated and down‐regulated genes in each plant (*x* axis, from left to right) in response to various hormones, abiotic stress, or light treatments (*y* axis) are displayed as heat maps by Genevestigator software. ABA, abscisic acid; GA, gibberellin; IAA, indole‐3‐acetic acid; R/FR, red/far‐red.
**Figure S9.** Functional categorization of the up‐regulated genes in each transgenic Arabidopsis plant. Up‐regulated genes (fold change > 2, FDR *P* < 0.05) in the transgenic plants were annotated by PageMan software. The numbers in each pie chart indicate the ratio against the total number of up‐regulated genes in each transgenic plant.
**Figure S10.** Functional categorization of the down‐regulated genes in each transgenic Arabidopsis plant. Down‐regulated genes (fold change < 0.5, FDR *P* < 0.05) in the transgenic plants were annotated by PageMan software. The numbers in each pie chart indicate the ratio against the total number of down‐regulated genes in each transgenic plant.
**Figure S11.** Map of sugar metabolism. The map was constructed based on the microarray data of the single‐ or double‐overexpressing plants analyzed by MapMan software. Squares and circles indicate the amounts of metabolites and transcripts, respectively. The expression level of the gene shows the highest or the lowest expression in each gene family. Yellow and blue colors show increased and decreased levels, respectively.
**Figure S12.** Expression analysis of the downstream genes of Arabidopsis PIF4 and auxin‐inducible genes in the single or double overexpressors. Expression levels of PIF4 downstream genes (a) and auxin‐inducible *SAUR* genes (b) in the transgenic plants analyzed by quantitative RT‐PCR. The plants were grown on agar medium for 2 weeks and harvested at ZT = 0. The error bars indicate the SD of more than 4 samples.

**Table S1.** Generation of *OsPIL1* and *DREB1A* double‐overexpressing Arabidopsis plants.

**Table S2.** Overlapping up‐regulated genes between the *35S*Ω:*DREB1A* plants and the double overexpressors.

**Table S3.** Overlapping up‐regulated genes between the *35S*Ω:*OsPIL1* plants and the double overexpressors.

**Table S4.** Overlapping down‐regulated genes between the *35S*Ω:*DREB1A* plants and the double overexpressors.

**Table S5.** Overlapping down‐regulated genes between the *35S*Ω:*OsPIL1* plants and the double overexpressors.

**Table S6.** Genes up‐regulated only in the double overexpressors.

**Table S7.** Genes down‐regulated only in the double overexpressors.

**Table S8.** Primer sequences used in this study.
