# Supplementary material for: Double overexpression of DREB and PIF transcription factors improves drought stress tolerance and cell elongation in transgenic plants
Source: Plant Biotechnol J. 2016 Nov 14;15(4):458–71. doi: 10.1111/pbi.12644 (PMC5362684; doi:10.1111/pbi.12644)
Supplement: Supplementary file 1 — Figure S1. Stability of the OsPIL1 protein in Arabidopsis protoplasts under light conditions. (a) Schematic diagram of the treatments used in the assay. Transfected protoplast suspensions were treated with 0.5% dimethyl sulfoxide (DMSO) or 50 μM MG132 under white light (50 ± 5 μmol photons/m2/s). (b) Stability of the OsPIL1 and PIF4 proteins. OsPIL1 and PIF4 proteins were expressed as sGFP fusion proteins under the control of the CaMV 35S promoter and the TMV Ω sequence. To normalize for transfection efficiency, 3 × Flag‐tag fused‐sGFP driven by the CaMV 35S promoter was co‐transfected into the protoplasts as an internal control. The levels of the fusion proteins were analyzed by immunoblotting using an antibody against GFP. Figure S2. Effect of co‐expressing both OsPIL1 and DREB1A on their individual transactivation activity in rice protoplasts. (a) Schematic diagram of the effector and reporter constructs used in the transactivation analysis of the rice protoplasts. The effector construct contains the maize ubiquitin promoter fused to the coding sequence of OsPIL1 or DREB1A. (b, c) Transactivation effects of DREB1A and OsPIL1 co‐expression. The reporter 12 × G box:GUS (b) or 3 × DRE:GUS (c) and the effectors were co‐transfected into rice protoplasts. To normalize for transfection efficiency, the luciferase (LUC) reporter gene driven by the maize ubiquitin promoter was co‐transfected as a control in each experiment. Bars show the SD of 4 replicates. The letters indicate significant differences among the assays (P < 0.01 according to Games‐Howell's multiple range test). Figure S3. Hypocotyl cell size of the single‐ or double‐overexpressing plants. (a) Cell surface in the hypocotyls of transgenic plants. The plants were grown on agar medium for 7 days. The cells were stained with toluidine blue. Bars = 50 μm. (b) Cell length calculated from the plants grown as in (a). The error bars show the SD of more than 50 cells in more than 4 seedlings (n > 200). The letters ind [file PBI-15-458-s004.doc]

**Supporting Information Figures**

**
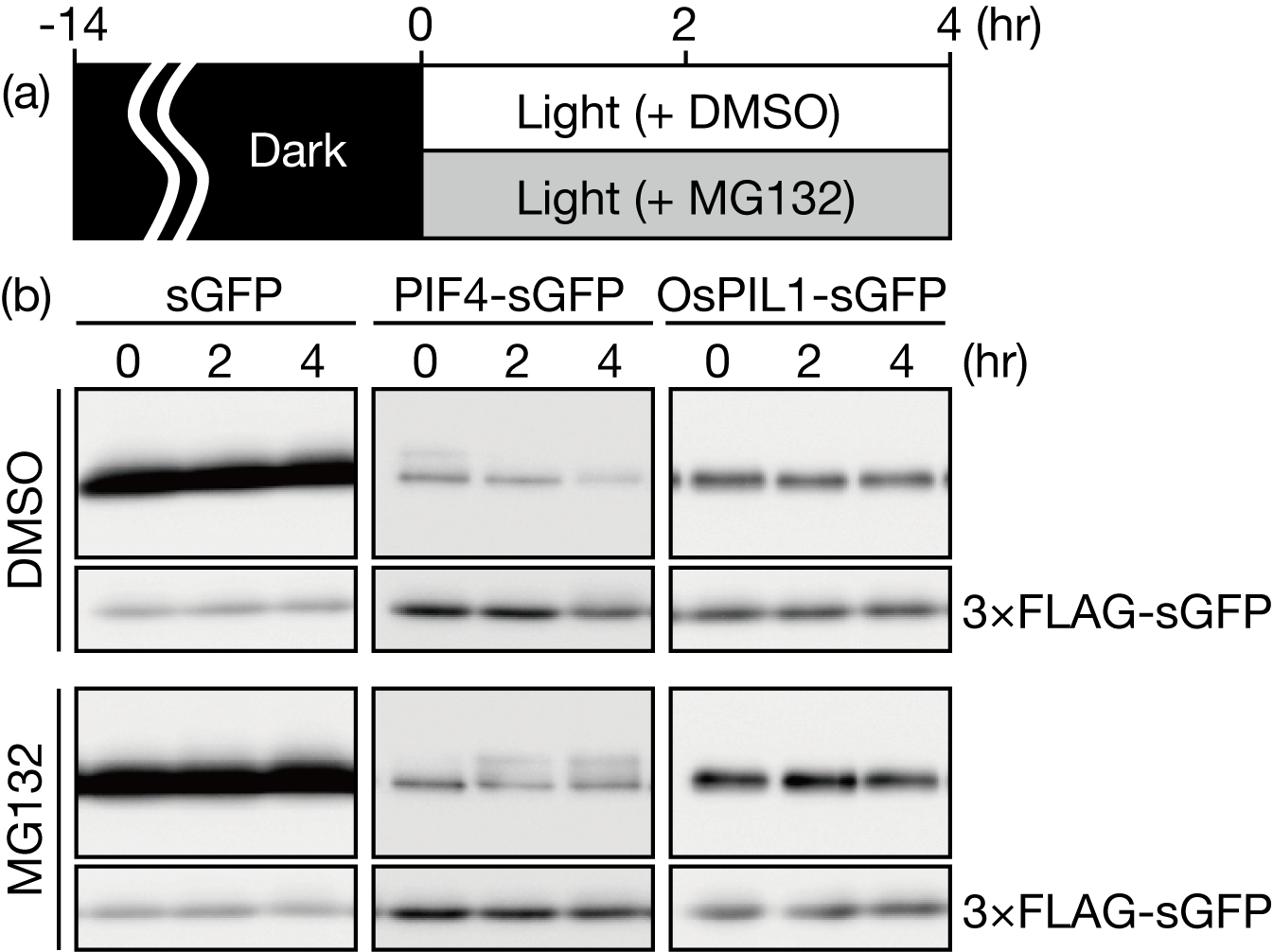
**

**Figure S1.** Stability of the OsPIL1 protein in Arabidopsis protoplasts under light conditions. (a) Schematic diagram of the treatments used in the assay. Transfected protoplast suspensions were treated with 0.5% dimethyl sulfoxide (DMSO) or 50 µM MG132 under white light (50 ± 5 µmol photons m^-2^ s^-1^). (b) Stability of the OsPIL1 and PIF4 proteins. OsPIL1 and PIF4 proteins were expressed as sGFP-fusion proteins under the control of the *CaMV 35S* promoter and the TMV Ω sequence. To normalize for transfection efficiency, 3×Flag-tag fused-sGFP driven by the *CaMV 35S* promoter was co-transfected into the protoplasts as an internal control. The levels of the fusion proteins were analyzed by immunoblotting using an antibody against GFP.


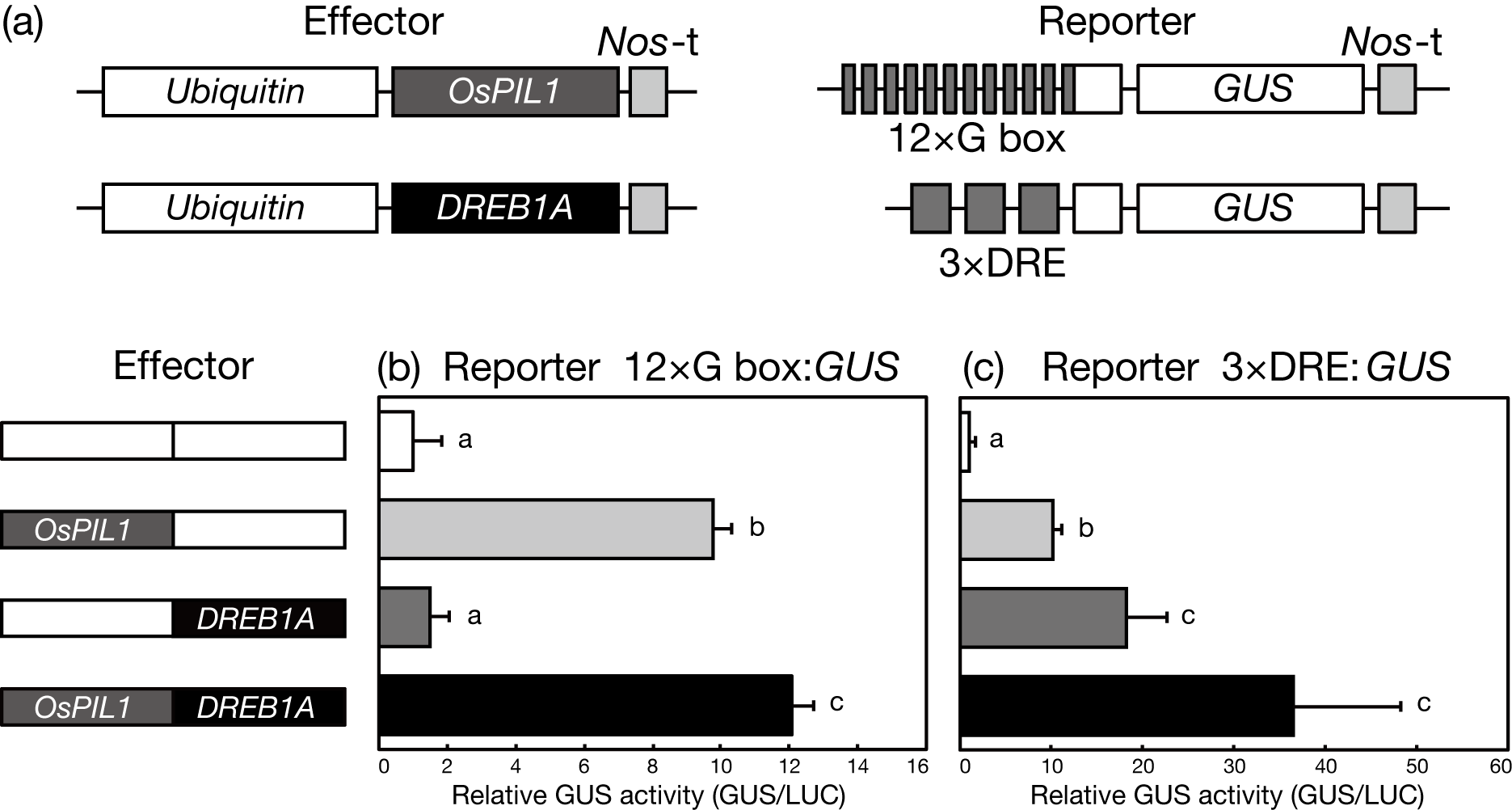


**Figure S2.** Effect of co-expressing both OsPIL1 and DREB1A on their individual transactivation activity in rice protoplasts. (a) Schematic diagram of the effector and reporter constructs used in the transactivation analysis of the rice protoplasts. The effector construct contains the *maize ubiquitin* promoter fused to the coding sequence of *OsPIL1* or *DREB1A*. (b-c) Transactivation effects of DREB1A and OsPIL1 co-expression. The reporter 12×G box:*GUS* (b) or 3×DRE:*GUS* (c) and the effectors were co-transfected into rice protoplasts. To normalize for transfection efficiency, the *luciferase* (*LUC*) reporter gene driven by the *maize* *ubiquitin* promoter was co-transfected as a control in each experiment. Bars show the SD of 4 replicates. The letters indicate significant differences among the assays (p < 0.01 according to Games-Howell’s multiple range test).


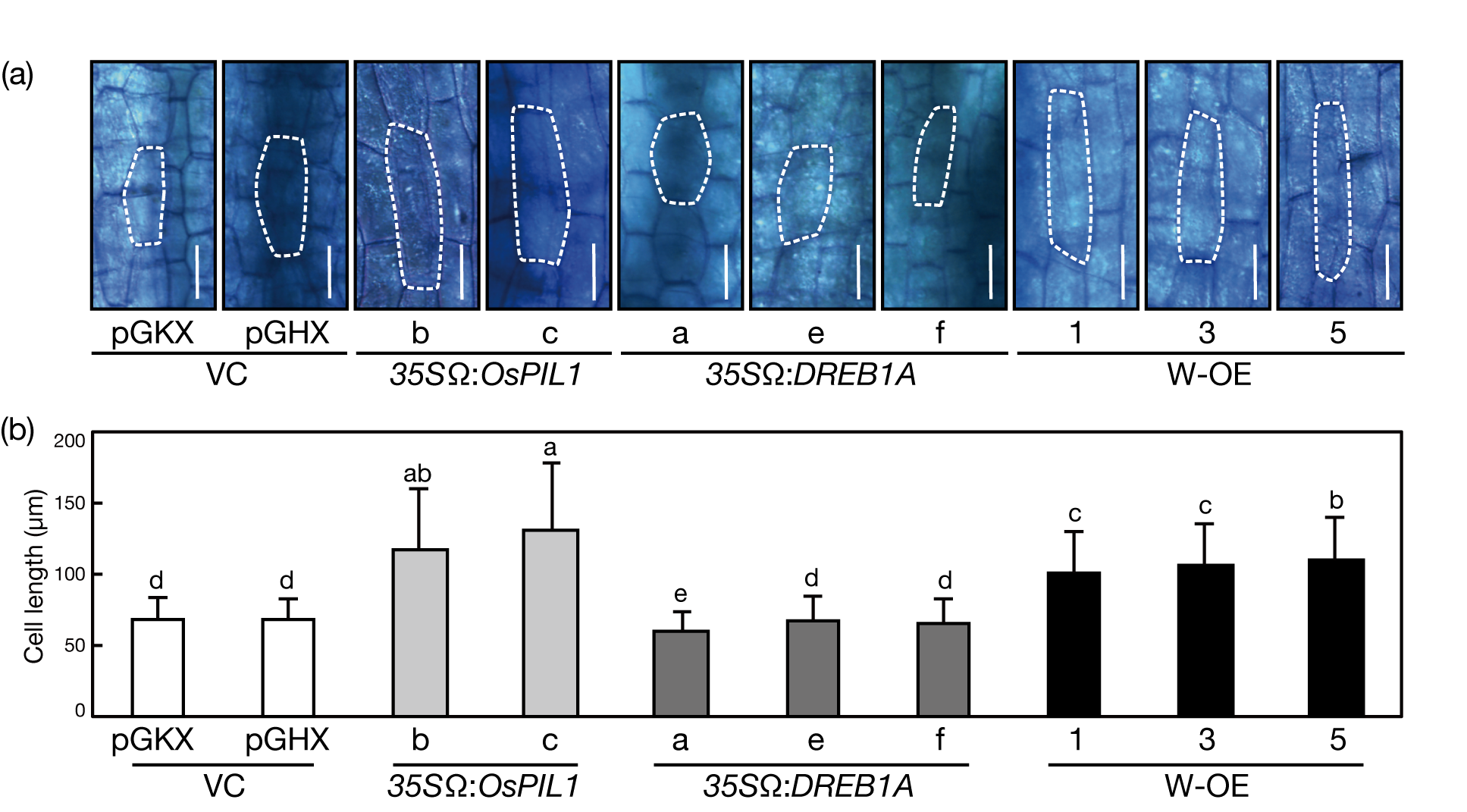
**Figure S3.** Hypocotyl cell size of the single- or double-overexpressing plants. (a) Cell surface in the hypocotyls of transgenic plants. The plants were grown on agar medium for 7 days. The cells were stained with toluidine blue. Bars = 50 μm. (b) Cell length calculated from the plants grown as in (a). The error bars show the SD of more than 50 cells in more than 4 seedlings (n > 200). The letters indicate significant differences among the seedlings (p < 0.01 according to Games-Howell’s multiple range test). VC and W-OE represent vector control and *OsPIL1* *DREB1A* double overexpressor, respectively.


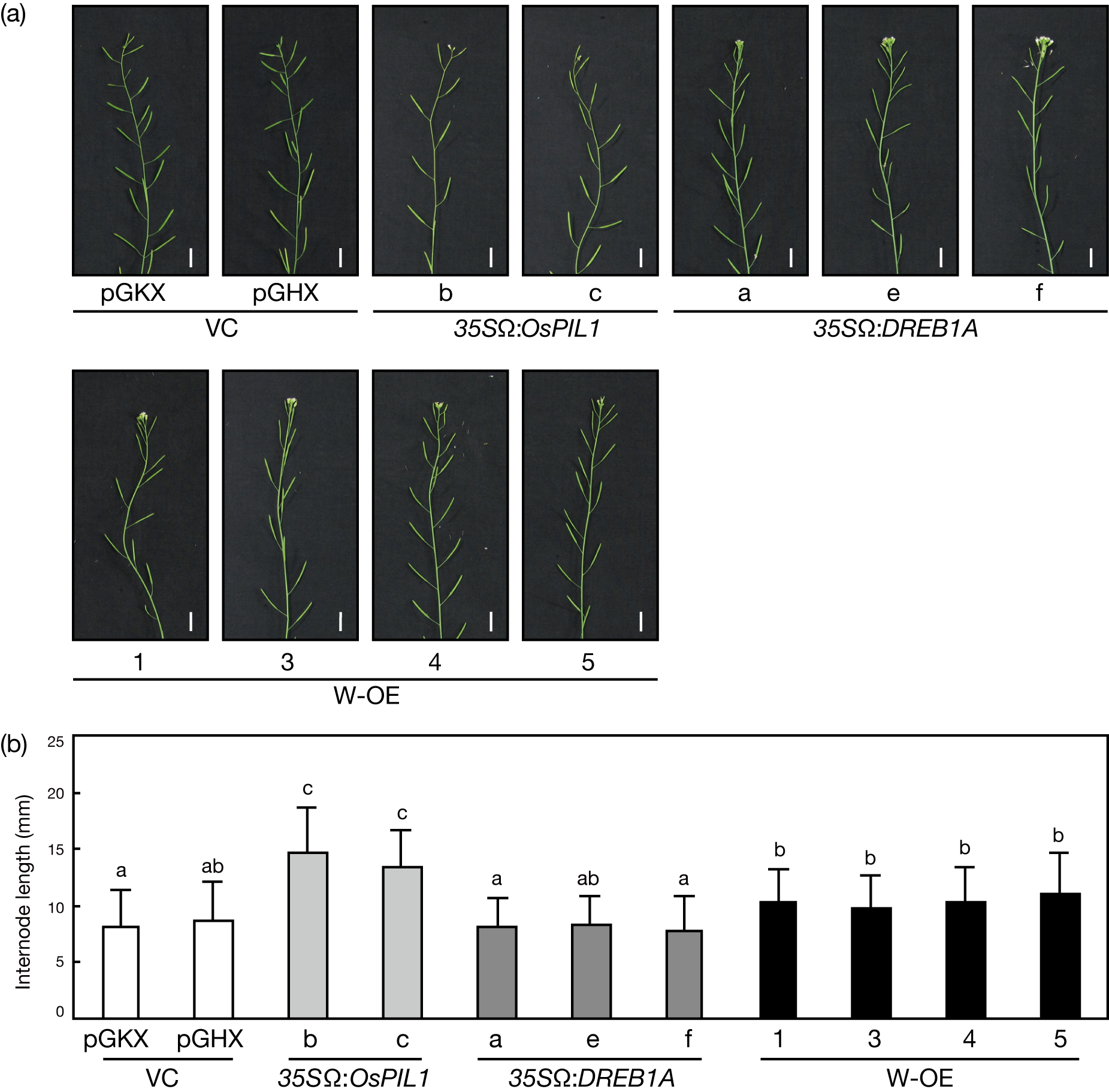
**Figure S4.** Internode length of the single- or double-overexpressing plants. (a) Stem morphology in 8-week-old transgenic plants. The plants were grown on agar medium for 2 weeks and then in soil pots for 6 weeks. Bars = 1 cm. (b) Internode length of the transgenic plants grown as in (a). The error bars show the SD of 10 internodes in 6 plants (n = 60). The letters indicate significant differences among the plants (p < 0.05 according to Games-Howell’s multiple range test).


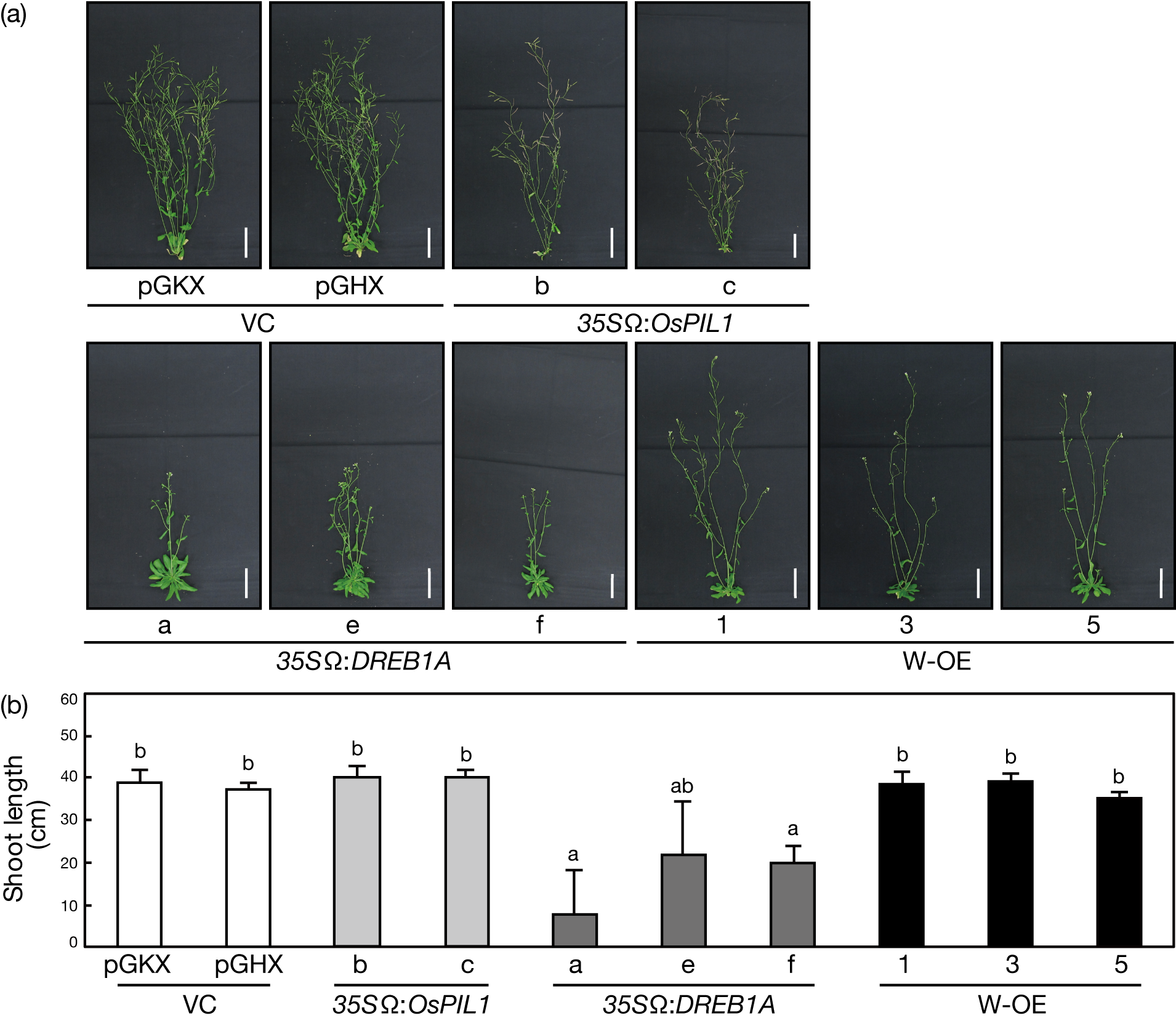
**Figure S5.** Shoot length of the single- or double-overexpressing plants. (a) Morphology of 8-week-old transgenic plants. The plants were grown on agar medium for 2 weeks and then in soil pots for 6 weeks. Bars = 10 cm. (b) Shoot length of the transgenic plants grown as in (a). The error bars show the SD of more than 4 plants. The letters indicate significant differences among the plants (p < 0.05 according to Games-Howell’s multiple range test).

**
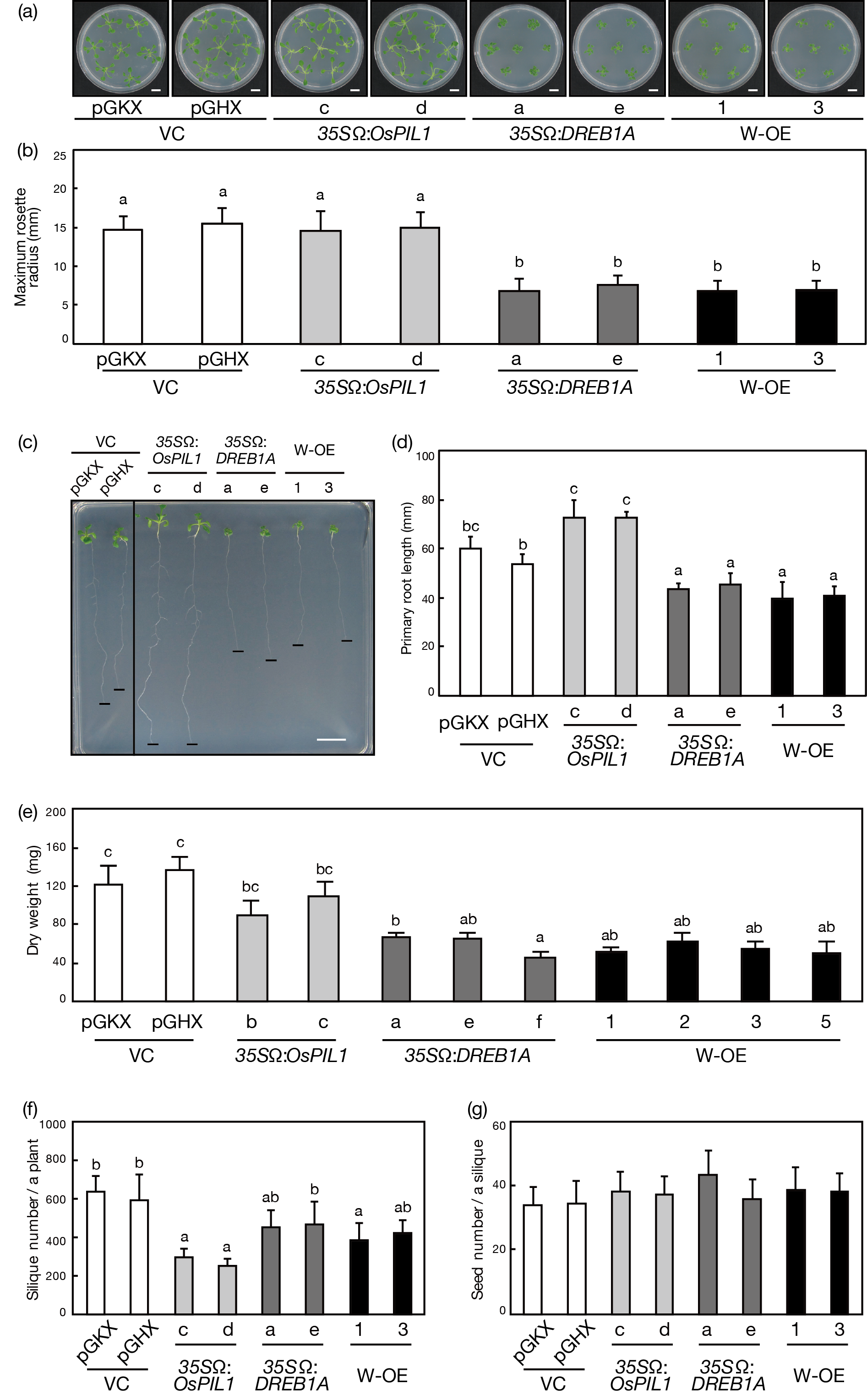
**

**Figure S6.** Growth of the single- or double-overexpressing plants. (a) Growth of 3-week-old transgenic Arabidopsis plants. The plants were grown on agar medium. Bars = 1 cm. (b) Average radius of the rosette calculated from the plants grown as in (a). The error bars show the SD of 14 plants. The letters indicate significant differences among the plants (p < 0.01 according to Tukey’s multiple range test). (c) Growth of 12-day-old transgenic Arabidopsis plants on vertically oriented agar medium. Black bars show the root apex of the transgenic plants. White bar = 1 cm. (d) Average primary root length of the transgenic plants grown as shown in (c). The error bars show the SD of more than 4 plants. The letters indicate significant differences among the plants (p < 0.05 according to Tukey’s multiple range test). (e) Average dry weight of 6-week-old transgenic Arabidopsis plants. The error bars show the SD of 6 plants. The letters indicate significant differences among the plants (p < 0.01 according to Games-Howell’s multiple range test). (f) Silique number in the transgenic Arabidopsis plants. The plants were grown on agar medium for 2 weeks and then in soil pots for more than 10 weeks. The error bars show the SD of more than 4 plants. The letters indicate significant differences among the plants (p < 0.05 according to Tukey’s multiple range test). (g) Seed number per silique for the transgenic plants grown as shown in (f). The error bars show the SD of 5 siliques in more than 4 plants (n > 20).


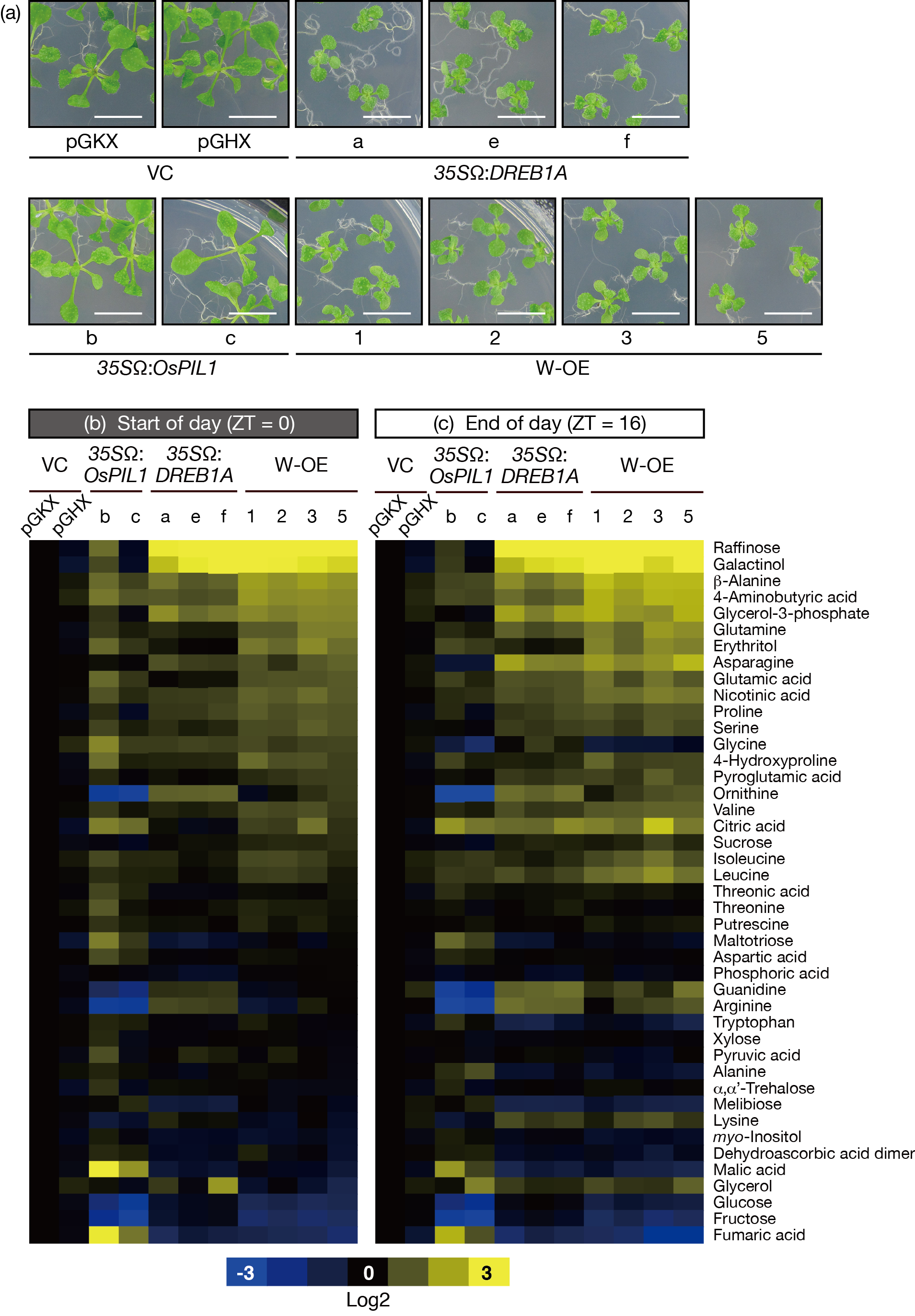


**Figure S7.** Comparison of the metabolite profiles in the transgenic plants at different sampling time points. (a) Growth of 2-week-old transgenic Arabidopsis plants. The plants were grown on agar medium. Bars = 1 cm. Metabolite profiles of the transgenic plants harvested at the start of the day (ZT = 0) (b) and the end of the day (ZT = 16) (c). The relative amounts of the metabolites are displayed as heat maps. Yellow and blue colors show increased and decreased levels, respectively.


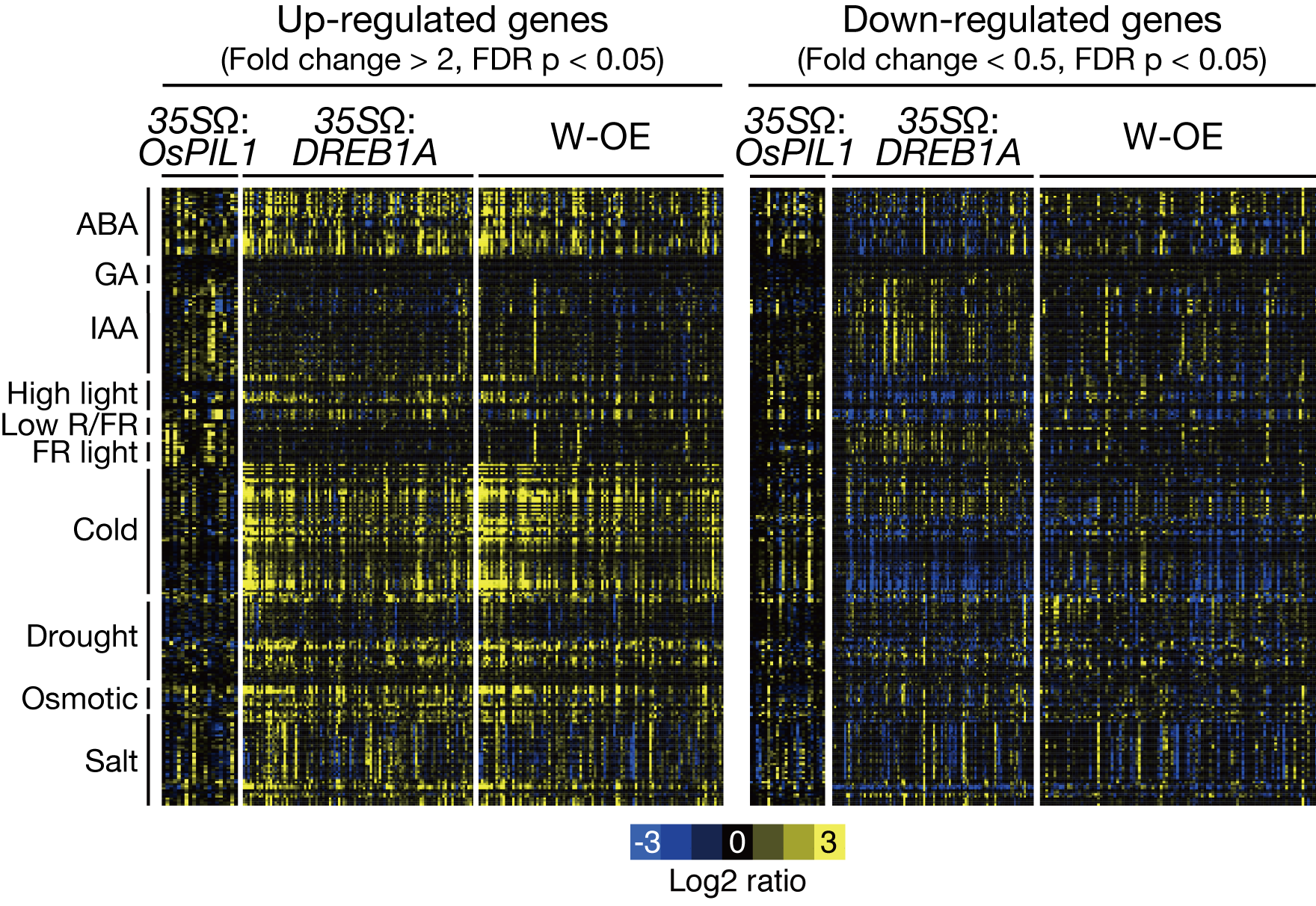
**Figure S8.** Hormone metabolism, abiotic stress, and light responses of the up-regulated and down-regulated genes in the single or double overexpressors. The expression ratios of the up-regulated and down-regulated genes in each plant (x axis, from left to right) in response to various hormones, abiotic stress, or light treatments (y axis) are displayed as heat maps by Genevestigator software. ABA, abscisic acid; GA, gibberellin; IAA, indole-3-acetic acid; R/FR, red/far-red.


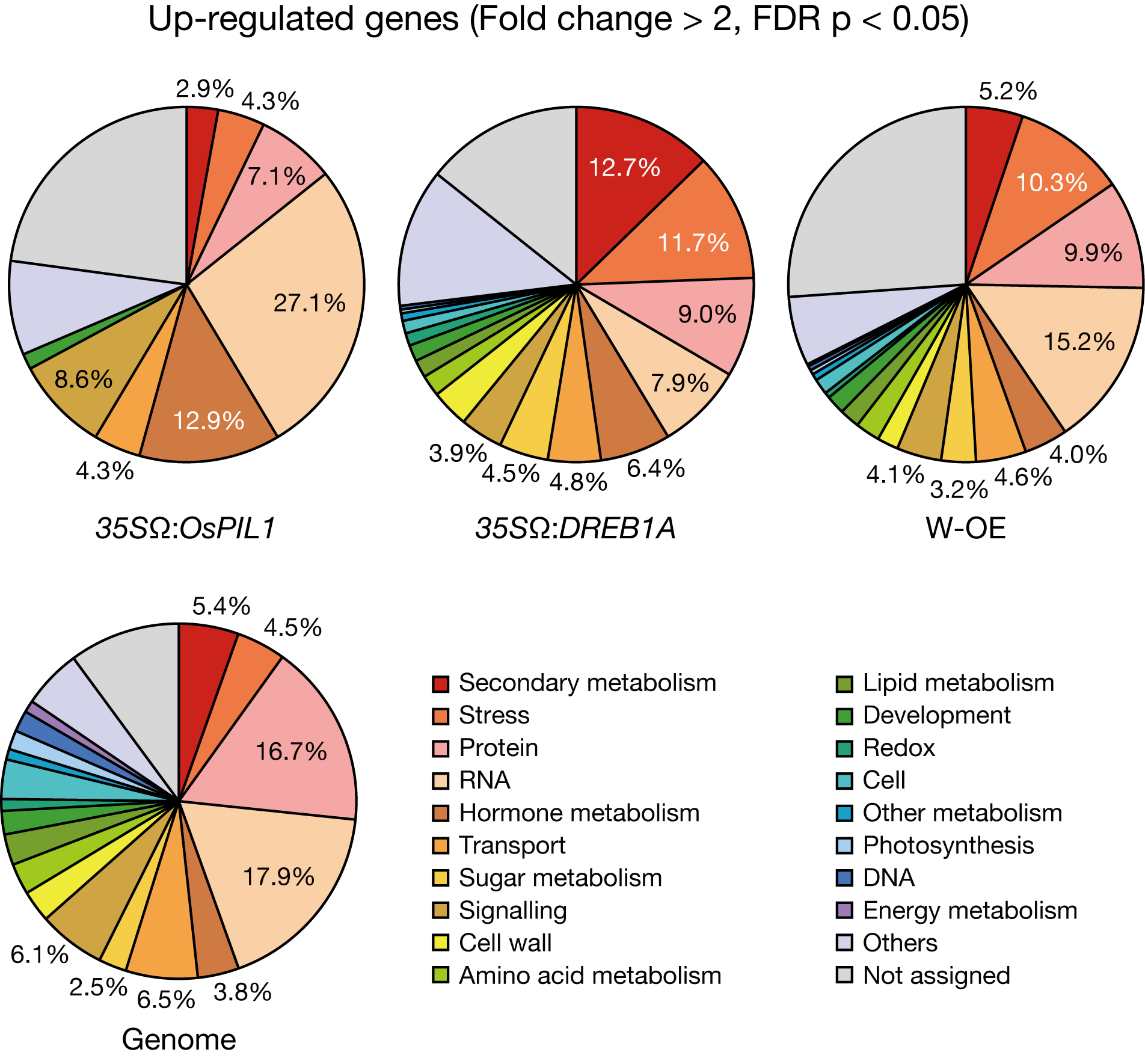
**Figure S9.** Functional categorization of the up-regulated genes in each transgenic Arabidopsis plant. Up-regulated genes (fold change > 2, FDR p < 0.05) in the transgenic plants were annotated by PageMan software. The numbers in each pie chart indicate the ratio against the total number of up-regulated genes in each transgenic plant.


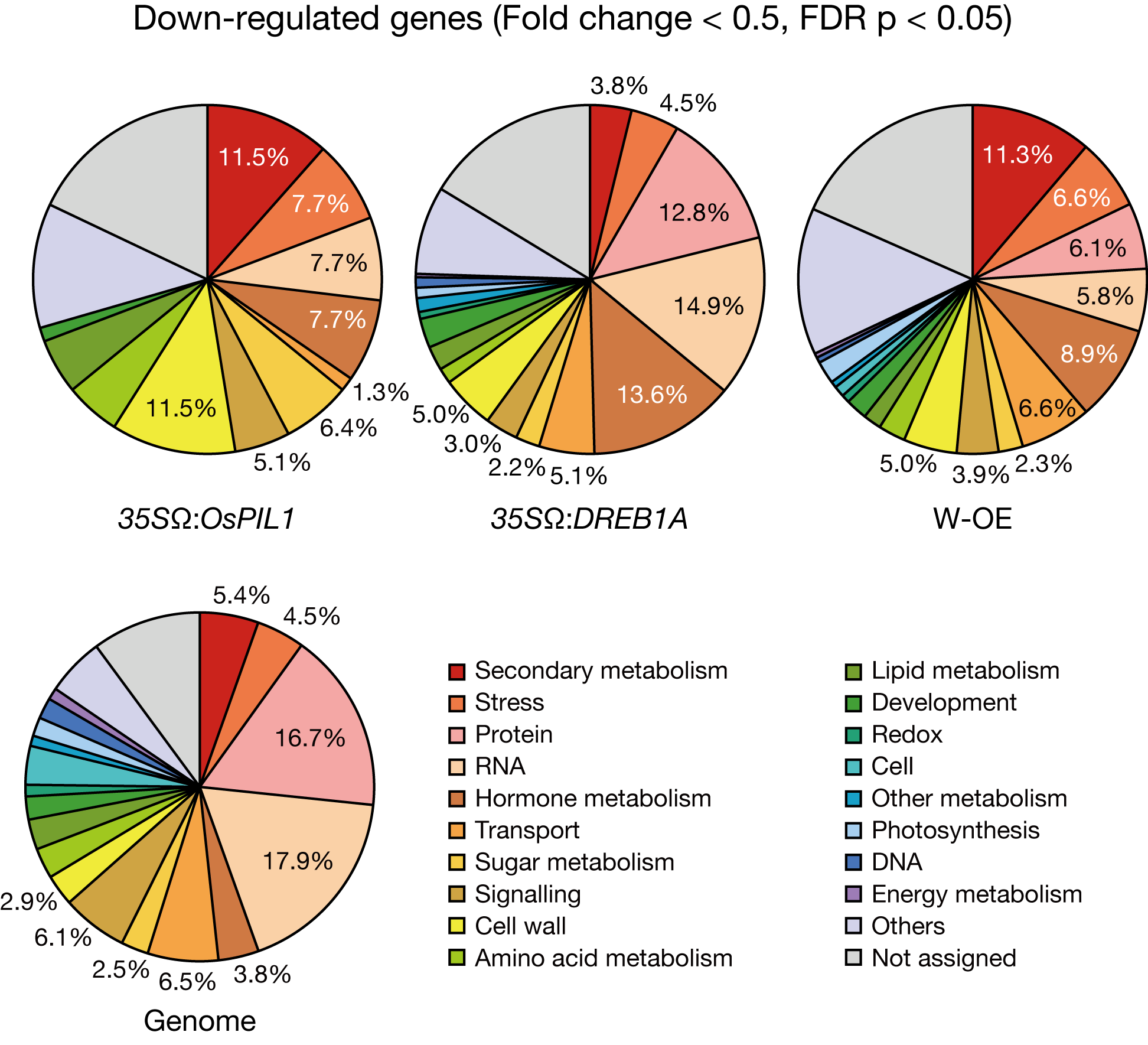
**Figure S10.** Functional categorization of the down-regulated genes in each transgenic Arabidopsis plant. Down-regulated genes (fold change < 0.5, FDR p < 0.05) in the transgenic plants were annotated by PageMan software. The numbers in each pie chart indicate the ratio against the total number of down-regulated genes in each transgenic plant.


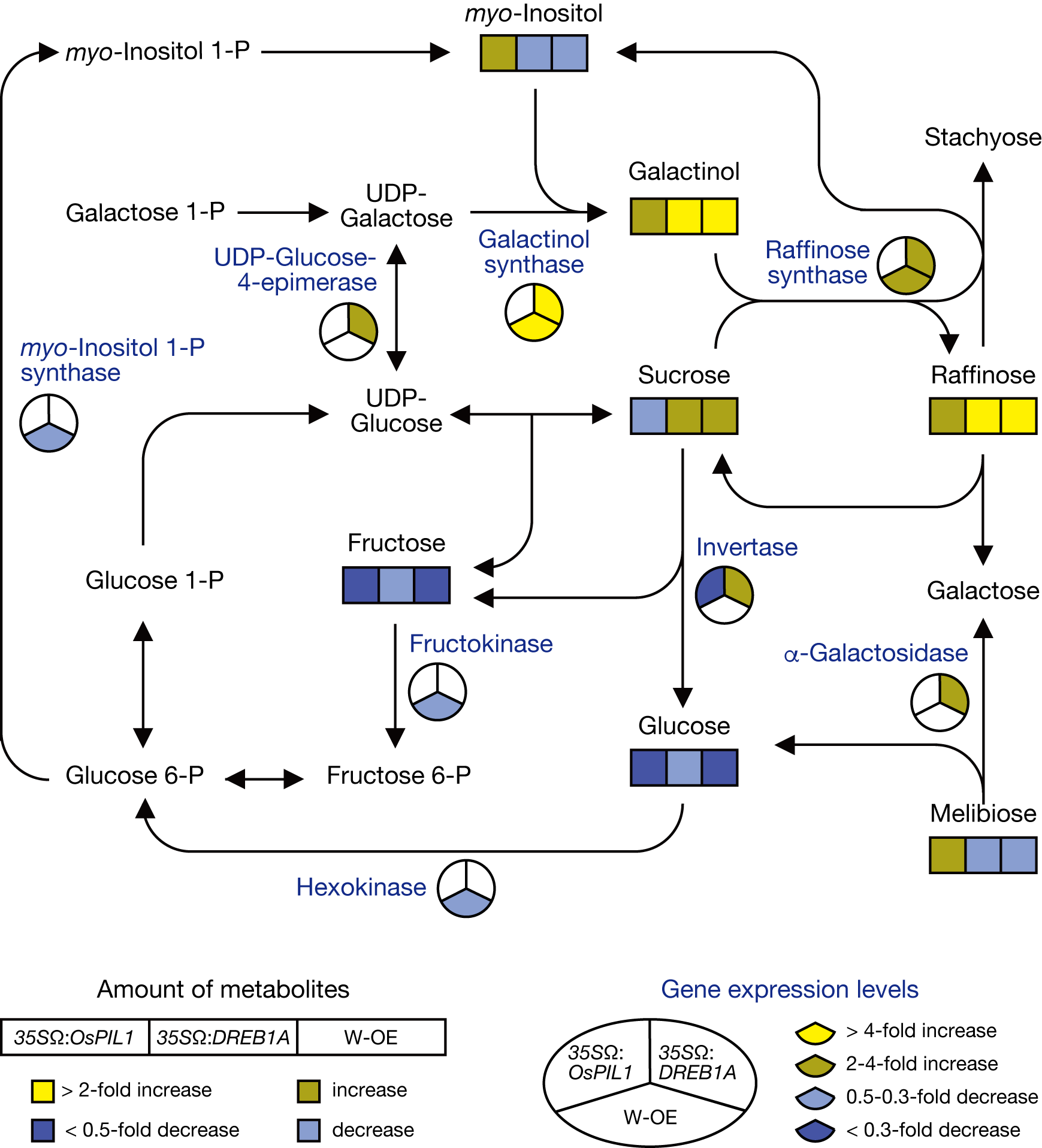
**Figure S11.** Map of sugar metabolism. The map was constructed based on the microarray data of the single- or double-overexpressing plants analyzed by MapMan software. Squares and circles indicate the amounts of metabolites and transcripts, respectively. The expression level of the gene shows the highest or the lowest expression in each gene family. Yellow and blue colors show increased and decreased levels, respectively.


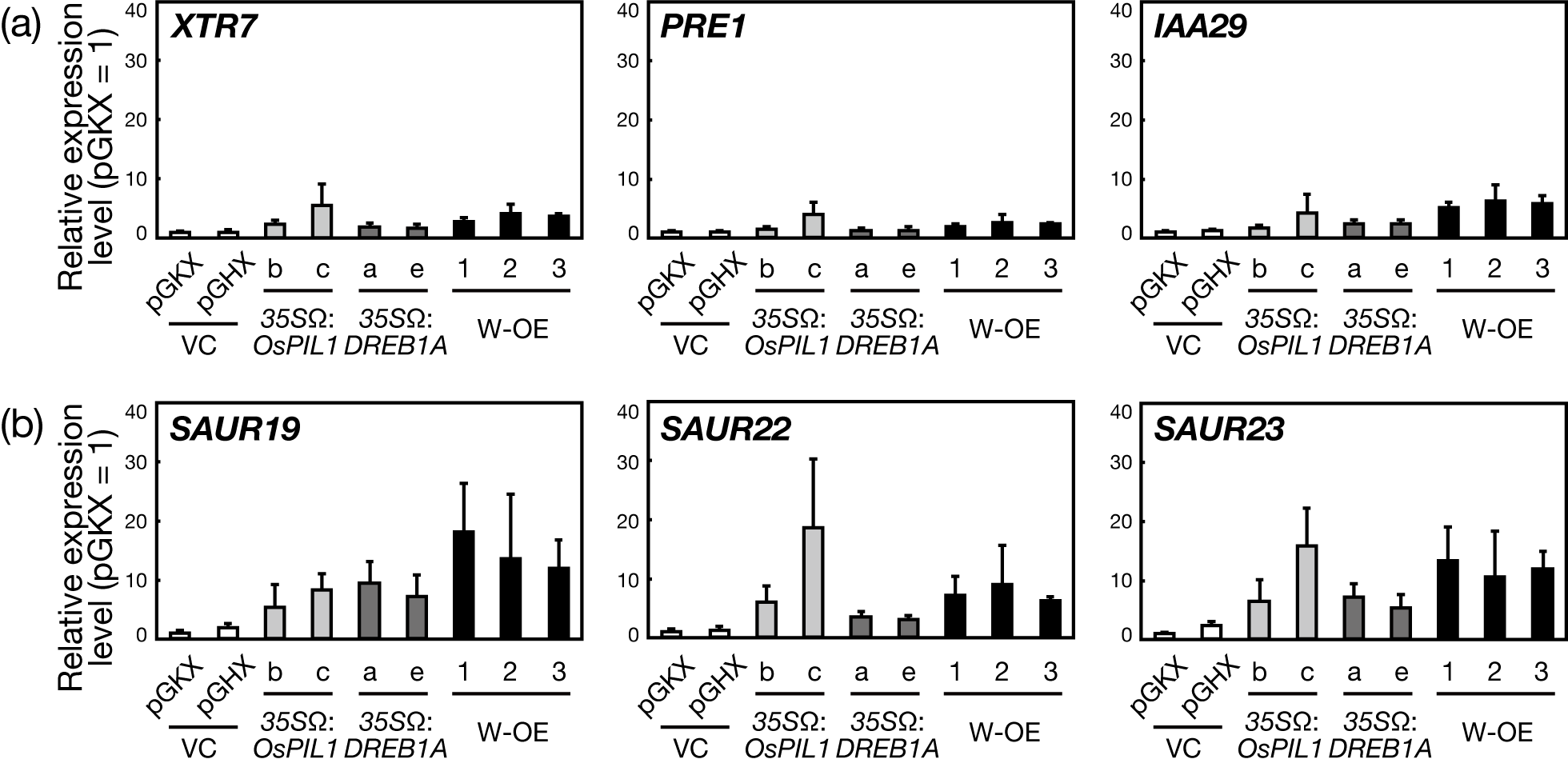
**Figure S12.** Expression analysis of the downstream genes of Arabidopsis PIF4 and auxin-inducible genes in the single or double overexpressors. Expression levels of PIF4 downstream genes (a) and auxin-inducible *SAUR* genes (b) in the transgenic plants analyzed by quantitative RT-PCR. The plants were grown on agar medium for 2 weeks and harvested at ZT = 0. The error bars indicate the SD of more than 4 samples.
