# Supplementary material for: Double overexpression of DREB and PIF transcription factors improves drought stress tolerance and cell elongation in transgenic plants
Source: Plant Biotechnol J. 2016 Nov 14;15(4):458–71. doi: 10.1111/pbi.12644 (PMC5362684; doi:10.1111/pbi.12644)
Supplement: Supplementary file 2 — Table S1. Generation of OsPIL1 and DREB1A double‐overexpressing Arabidopsis plants. [file PBI-15-458-s007.doc]

**Table S1.** Generation of *OsPIL1* and *DREB1A* double-overexpressing Arabidopsis plants.

| *OsPIL1* and *DREB1A* double overexpressing Arabidopsis plants | Female parent | Male parent |
| --- | --- | --- |
| W-OE-1 | *35S*Ω:*DREB1A*-a | *35S*Ω:*OsPIL1*-c |
| W-OE-2 | *35S*Ω:*DREB1A*-e | *35S*Ω:*OsPIL1*-b |
| W-OE-3 | *35S*Ω:*DREB1A*-e | *35S*Ω:*OsPIL1*-c |
| W-OE-4 | *35S*Ω:*DREB1A*-e | *35S*Ω:*OsPIL1*-h |
| W-OE-5 | *35S*Ω:*DREB1A*-f | *35S*Ω:*OsPIL1*-b |
