# Supplementary material for: Double overexpression of DREB and PIF transcription factors improves drought stress tolerance and cell elongation in transgenic plants
Source: Plant Biotechnol J. 2016 Nov 14;15(4):458–71. doi: 10.1111/pbi.12644 (PMC5362684; doi:10.1111/pbi.12644)
Supplement: Supplementary file 9 — Table S8. Primer sequences used in this study. [file PBI-15-458-s005.doc]

**Table S8** Primer sequences used in this study.

| Name | Sequence (5'-3') |
| --- | --- |
| 18S_rRNA_F | AAACGGCTACCACATCCAAG |
| 18S_rRNA_R | CCTCCAATGGATCCTCGTTA |
| OP1transgene_rtP_5/n | CAAACAGTGCCACCACAGGT |
| OP1_CDS_3/c | CTAAATTCCATCAGAGGTTGG |
| DREB1A_436F | CGCTGACTCGGCTTGGA |
| DREB1A_543R | GCATCACACATCTCATCCTGAAAC |
| HFR1_F | TAAATTGGCCATTACCACCGTTTA |
| HFR1_R | ACCGTGAAGAGACTGAGGAGAAGA |
| PIL1_rt5' | ATGCACCACCACAAATGCTA |
| PIL1_rt3' | ACGCAGACTTTGGGAATTGA |
| ATHB2-F | GAGGTAGACTGCGAGTTCTTACG |
| ATHB2-R | GCATGTAGAACTGAGGAGAGAGC |
| RD29A_qRT_F | CTTGATGGTCAACGGAAGGT |
| RD29A_qRT_R | CAATCTCCGGTACTCCTCCA |
| COR15A_400F | GAAAAAAACAGTGAAACCGCAGAT |
| COR15A_463R | CCACATACGCCGCAGCTT |
| GolS3_qRT_F | ACAGGCCAAGAAGGAAATATGG |
| GolS3_qRT_R | GATGGAGCTTTGGCACATTG |
| IAA6_F_461-484 | AATCTCTTCGGCTGTCTTGGCATA |
| IAA6_R_645-624 | TGGAGACCAAAACCAGTTGCAT |
| IAA19_332-351_F | TGTAAGGAAGCTTCGACCAC |
| IAA19_501-481_R | TCTTTCAAGGCCACACCGATG |
| IAA1_266-287_F | GCTCCTCCTCCTGCAAAAACAC |
| IAA1_322-301_R | ACGGTTAGATCTCACTGGAGGC |
| EXPA1_F_276-295 | AAGGCTATGGAACCAACACG |
| EXPA1_R_448-429 | GTTGTTCGGTAAGGCGTTGT |
| EXPA8_F_140-159 | TTCCTCCAAGGAACTCATGG |
| EXPA8_R_392-373 | CTGTGACGGTGATGGTTGAC |
| FT_F_236-252 | ATGGTGGATCCAGATGTTCC |
| FT_R_464-483 | TACACTGTTTGCCTGCCAAG |
| MIR172A_F_558-577 | GACTAATTTCCGGAGCCACG |
| MIR172_R_703-684 | TAGTCGTTGATTGCCGATGC |
| XTR7_qRT-PCR_750_F | CGGCTTGCACAGCCTCTT |
| XTR7_qRT-PCR_828_R | TCGGTTGCCACTTGCAATT |
| PRE1_F_338-410 | GTTCTGATAAGGCATCAGCCTCG |
| PRE1_R_548-526 | CATGAGTAGGCTTCTAATAACGG |
| IAA29_RT_F | ATCACCATCATTGCCCGTAT |
| IAA29_RT_R | ATTBCCACACCATCCATCTT |
| SAUR19_F_49-77 | CTTCAAGAGCTTCATAATAATTCAAACTT |
| SAUR19_R_397-373 | GAAGGAAAAAATGTTGGATCATCTT |
| SAUR22_F_73-100 | GACAAATAGAGAATTATAAATGGCTCTG |
| SAUR22_R_412-385 | ATGAATTAAGTCTATATCTAACTCGGAAA |
| SAUR23_F_54-80 | ATTCAAACTTTCAGACAAAAGAAATGG |
| SAUR23_R_401-376 | ACAAGGAAACAACTCTATCTCTAACT |
